# Microglia-Astrocyte Communication in Alzheimer’s Disease

**DOI:** 10.3233/JAD-230199

**Published:** 2023-09-26

**Authors:** Yingying Wu, Ulrich L.M. Eisel

**Affiliations:** a Department of Molecular Neurobiology, Groningen Institute for Evolutionary Life Sciences (GELIFES), University of Groningen, Groningen, The Netherlands; b Department of Neurology, The Second Affiliated Hospital of Hainan Medical University, Haikou, Hainan, China

**Keywords:** Alzheimer’s disease, astrocyte, cellular crosstalk, microglia, neuroinflammation

## Abstract

Microglia and astrocytes are regarded as active participants in the central nervous system under various neuropathological conditions, including Alzheimer’s disease (AD). Both microglia and astrocyte activation have been reported to occur with a spatially and temporarily distinct pattern. Acting as a double-edged sword, glia-mediated neuroinflammation may be both detrimental and beneficial to the brain. In a variety of neuropathologies, microglia are activated before astrocytes, which facilitates astrocyte activation. Yet reactive astrocytes can also prevent the activation of adjacent microglia in addition to helping them become activated. Studies describe changes in the genetic profile as well as cellular and molecular responses of these two types of glial cells that contribute to dysfunctional immune crosstalk in AD. In this paper, we construct current knowledge of microglia-astrocyte communication, highlighting the multifaceted functions of microglia and astrocytes and their role in AD. A thorough comprehension of microglia-astrocyte communication could hasten the creation of novel AD treatment approaches.

## ALZHEIMER’S DISEASE

Alzheimer’s disease (AD), a very common progressive neurodegenerative disorder, is generally categorized as early-onset AD (prior to age 65) and late-onset AD (65 years or more). In the clinic, AD is perceived as a disease continuum consisting of three phases: preclinical AD, mild cognitive impairment (MCI), and dementia [1]. Although individuals with preclinical AD have yet to develop symptoms, biomarker testing shows measurable brain changes, including decreased cerebrospinal fluid and plasma amyloid-β (Aβ), increased global signal on amyloid positron emission tomography (PET) scans, as well as early neuroinflammatory changes (such as microgliosis as detected by PK11195 PET imaging [2]). People with MCI due to AD show subtle symptoms, for example, memory and thinking problems that do not impair their ability to perform during day-to-day activities. Besides that, they have AD-related biomarker changes in the brain. The hallmark of AD dementia is biomarker evidence of AD brain changes in addition to noticeable memory, thought, or behavioral symptoms that interfere with everyday activities. Since AD affects people in different ways, each person may have different symptoms or progress differently through the stages.

Glenner and Wong [3] initially identified the Aβ peptide as a primary component of meningovascular amyloid in 1984, and then Masters and co-authors [4] identified the Aβ peptide as an essential constituent of Aβ plaques in 1985. Similarly, tau was first demonstrated to represent the cause of AD in 1988 [5]. As first observed over 100 years ago, the presence of intracellular accumulation of neurofibrillary tangles (NFTs) formed by hyperphosphorylated tau and senile plaques made of extracellular Aβ peptides as the key pathological features of AD are required for diagnosis [6]. The Aβ accumulation and NFTs lead to synaptic and neuronal loss. The degree of neuronal loss in the brain, especially in the hippocampus and the cerebral neocortex, is considered to be involved in the clinical manifestation of AD. In AD, it is reported that the reduction of the number of neurons is moderate in cortical structures (26–30%), while the reduction in the number of pyramidal neurons is up to 45%, which correlates with the density of NFTs and senile plaques [7–10]. Cortical atrophy is a result of neuronal loss and typically starts at the mesial temporal lobe. On macroscopic inspection, it is possible to determine that AD is present because of the obvious features of gross brain shrinkage and the loss of neuromelanin pigmentation in the locus coeruleus.

Over the past few years, the tau and amyloid hypotheses have become the dominant fundamental hypotheses for explaining pathogenic mechanisms. However, an emerging amount of literature confirms the opinion that inflammation is the principal player orchestrating the pathophysiology of AD. Examples include increased production of pro-inflammatory cytokines in the central nervous system (CNS), especially by microglia and astrocytes [11]. The neuroinflammatory response is responsible for the “two-edged sword” effect in AD. Neuroinflammation, in the early stages of AD, serves as a self-defense mechanism to safeguard the brain by accelerating tissue repair and the rapid removal of potentially damaging stimuli. As the disease continues to advance, however, an ongoing inflammatory response results in adverse outcomes, fueling neurodegeneration [12, 13]. Considering that immune dysfunction starts early in the disease course, perhaps even before relevant pathogenic brain changes, it is significant to underline the neuroinflammatory processes are not confined to the brain alone [14–21]. The major immune component of the intact CNS is composed of glia, primarily microglia, and astrocytes, involves tight and fine-tuned crosstalk, and acts as a main actor in the ongoing neuroinflammatory response in AD [22]. Strikingly, activated microglia and reactive astrocytes are especially detected in high numbers in close proximity to senile plaques in the AD brain, indicating their crucial implication in the pathogenesis of AD [23–25].

## MICROGLIA IN THE CNS

Microglia, the brain-resident macrophages, make up about 10%–15% of the total adult CNS cells. They are predominately located within the gray matter than the white matter, with the basal ganglia, hippocampus, olfactory telencephalon, and substantial nigra having the highest levels [26, 27]. Despite being extensively studied, the origin of microglia remains a subject of debate. The early 21st century was definitively defined [28] by the description in the 1990 s [29, 30] that microglia originated from primitive yolk sac macrophages.

In physiological conditions, microglia are considered to be in a resting or quiescent state, they represent a ramified phenotype characterized by long branching processes and a small cellular body [31]. A major function of resting microglia is that they constantly and vigilantly surveil the cerebral parenchyma for any changes in brain homeostasis that may occur using their dynamic and motile cellular processes as sentinels [32–34]. Microglia undergo a remarkable transformation from their stationary to an active state in response to certain cues, such as brain injury [35] or immunological stimuli, and adopt a less ramified morphology and a more amoeboid morphology as their soma grows and their cellular processes shorten [36]. At the site of the lesion, activated microglia start the processes necessary for tissue repairs, such as the phagocytosis of pathogens and the removal of cellular debris and degenerated cells [37, 38].

**Fig. 1:**
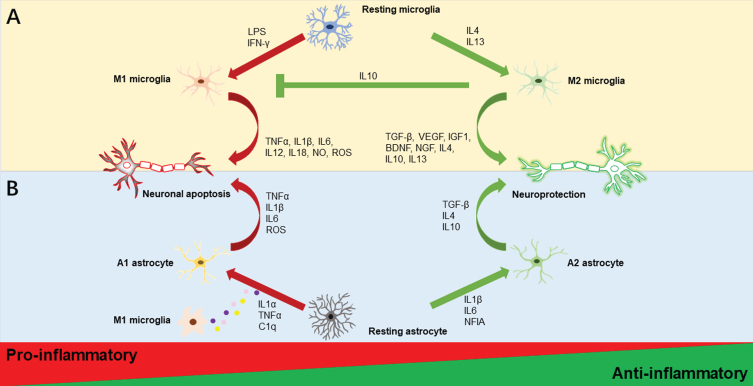
**Illustrations of microglia and astrocyte polarization.** Activated microglia are often classified as M1 or M2 phenotypes as displayed in the upper half of Fig. 1. Resting microglia polarize to the M1 phenotype and produce pro-inflammatory substances such as TNF*α*, IL1β, IL6, IL12, IL18, NO, and ROS, when LPS and IFN-*γ* are present. In contrast, IL4 and IL13 stimulation causes M2 polarization, which increases the secretion of anti-inflammatory substances such as TGF-β, VEGF, IGF1, BDNF, NGF, IL4, IL10, and IL13. Additionally, M2 microglia could promote the inhibition of M1 microglia by the anti-inflammatory cytokine IL10. Activated astrocytes are usually divided into A1 and A2 phenotypes as depicted in the lower half of Figure 1. Astrocytes may transform into different reactive astrocyte phenotypes depending on the stimulus. The M1 microglia’s production of the pro-inflammatory cytokines IL1*α*, TNF*α*, and C1q causes the A1 neurotoxic phenotype and encourages the secretion of TNF*α*, IL1β, IL6, and ROS. Meanwhile, IL1β, IL6, and NFIA trigger the A2 phenotypic change with neuroprotective effects that increase anti-inflammatory molecules TGF-β, IL4, and IL10. BDNF, brain-derived neurotrophic factor; C1q, complement component 1q; IFN-*γ*, interferon-gamma; IGF1, insulin-like growth factor 1; IL, interleukin; LPS, lipopolysaccharide; NFIA, nuclear factor IA; NGF, nerve growth factor; NO, nitric oxide; ROS, reactive oxygen species; TGF-β, transforming growth factor-beta 1; TNF-*α*, tumor necrosis factor-alpha; VEGF, vascular endothelial growth factor.

An extensive body of *in vitro* studies has classically described microglial activation states as being either classical activation (M1, “pro-inflammatory”) or alternative activation (M2, “anti-inflammatory”) on the basis of the pattern of cytokine secretion or alterations in cellular gene expression. In general, M1 microglia is characterized by excessive production of a variety of potentially harmful pro-inflammatory mediators, such as interleukin-1β (IL1β), IL6, IL12, IL18, tumor necrosis factor-*α* (TNF*α*), nitric oxide (NO), and prostaglandins, and are relatively poor phagocytes, resulting in an exacerbation of inflammation. These characteristics are driven by interferon *γ* (IFN-*γ*) and lipopolysaccharide (LPS) stimulation, according to evidence from *in vitro* investigations [39, 40]. In contrast, IL4 or IL13-induced M2 microglia are characterized by cellular debris clearance and the important release of numerous trophic factors, including growth and neurotropic factors like brain-derived neurotrophic factor (BDNF), insulin-like growth factor-1 (IGF1), nerve growth factor, transforming growth factor-β (TGF-β), and vascular endothelial growth factor [39, 40] (see Fig. 1A). M2 microglia can be further characterized into subcategories, M2a, M2b, and M2c based on their distinctive profiles of pro-inflammatory cytokines. M2a microglia, considered to be the alternatively activated microglia, is associated with neuroinflammation resolution and phagocytosis; M2b microglia, also known as the type II alternative activated microglia, is related to the increased phagocytic and immunomodulatory activity; M2c microglia referred to as the acquired deactivated microglia, is involved in anti-inflammatory actions [41].

Various CNS injury model studies illustrate that most newly recruited microglia at the injured site are the M2 phenotype in the early stages, but gradually switch to the M1 phenotype approximately a week after the injury [42–46]. This phenotype shifts from M2 to M1 resulting in the exacerbation of the inflammatory response. However, the M1-M2 dichotomy for categorizing the microglial phenotype is an oversimplification as it fails to recapitulate fully microglial functions. For instance, microglia expressed M1 and M2 phenotypic markers in the same cell across multiple time points in the context of traumatic brain injury [47]. More importantly, M2 microglia are not always beneficial [48]. An example can be seen in the work undertaken by Chakrabarty et al. [49] where exacerbated Aβ deposition was observed in the TgCRND8 mice injected with adeno-associated virus serotype 1 expressing murine IL4 in the CNS. As their findings were in conflict with other published *in vivo* studies [50], the authors claimed that the increase in amyloid pathology was caused by the inability of microglia to successfully clear Aβ [49].

Recently, a novel subset of microglia known as disease-associated microglia (DAM), a fraction of microglia with a distinctive transcriptional and functional signature, has been observed in immune cells of the CNS of neurodegenerative diseases, including AD [51]. DAM is identified molecularly as immune cells that display the typical microglial markers Iba1, Cst3, and Hexb, together with the upregulation of “neurodegeneration” genes, including numerous recognized AD risk genes (e.g., Apoe, Lpl, Trem2, Tyrobp, and Ctsd), and the downregulation of “homeostatic” gene set (e.g., P2ry12/P2ry13, Cx3cr1, Cst3, Cd33, Csf1r, and Tmem119) [51–53]. It should be noted that DAM cells grow in number as amyloidosis progresses, are located in close proximity to amyloid plaques, and exhibit signs of Aβ uptake. Lysosomal, phagocytosis, lipid metabolism, and immune response pathways are highlighted by DAM gene analytics. In the first stage of DAM activation, which is independent of, the triggering receptor expressed on myeloid cells 2 (TREM2), microglia engage and negatively regulate inhibitory receptors. The second stage, which is dependent on TREM2 and is required for full phagocytic capacity, appears to occur after the first stage [51]. The stage 1 DAM transition is necessary for the subsequent activation of the stage 2 DAM program; however, it is yet unclear how microglia move from stage 1 to stage 2 and activate the expression of TREM2 [51, 54–56]. It is interesting that one subtype of microglia seems to be beneficial for AD. The inconsistent evidence regarding microglia activation, phagocytosis, Aβ clearance, and the toxic versus beneficial effects attributed to microglia in AD could be explained in part by the existence of a microglia subtype showing beneficial impacts on the development of the disease [56].

## THE BIPHASIC ROLE OF MICROGLIA IN AD

Inflammatory markers have been consistently detected in AD brains for many decades. Microglia, which represent the major source of inflammatory factors have been determined to perform an essential role in orchestrating neuroinflammation. Inflammatory substances generated by microglia and astrocytes may harm nearby tissues and, when combined with disease-associated molecular patterns (DAMPs) that have been released, may aggravate neuroinflammation and activate glia, resulting in a vicious cycle of neuroinflammation. The effects of chronic neuroinflammation on the CNS can be severe, including synapse loss, cognitive impairment, and overt neurodegeneration [57–60]. It is possible that this shift away from reparative reactions may be the result of an M2 that does not respond efficiently. Since fewer M2 microglia result in lower amounts of neuroprotective substances such as IGF1 and BDNF, which are produced by microglia, the absence of M2 cells can also make it more difficult for neuroinflammation to be regulated. Therefore, a key factor driving neurodegeneration may be the absence of a proper M2 reaction [48].

## MICROGLIA AND Aβ

The first groundbreaking finding concerning microglial involvement in the progression of AD was published at the beginning of the 1990 s reporting that microglia were closely related to Aβ plaques in the brains of people who have AD [61, 62]. In most cases, microglia focally aggregate around the dense-core plaques in human postmortem brain tissue slices. Some are also observed in clusters adjacent to diffuse plaques [63, 64]. Numerous studies have shown that Aβ itself attracts microglia in both human samples [65, 66] and mouse transgenic models of AD [67–70], which may reflect the interaction between Aβ and both microglia and astrocytes, stimulating chemokines secretion [71]. From *in vivo* imaging investigations, it is evident that plaque development is extremely rapid, and microglia react to Aβ plaques by extending their processes and migrating toward the initial plaque shortly after it forms [72, 73]. The entire amyloid surface area is extensively covered by microglial processes, but some of them have less microglial coverage. These plaques are the ones that have tended to increase in volume in the course of a month [73]. Additionally, there was a highly proportional correlation between microglia and Aβ plaques, with the number and size of microglia changing as the size of plaques did, regulating plaque dynamics [73].

Meanwhile, microglia depletion studies have demonstrated that microglia contribute to plaque formation, compaction, and growth, neuritic dystrophy mitigation, and hippocampal neuronal gene expression regulation in response to Aβ pathology, implicating the link between microglia and the development and progression of various aspects of AD [74, 75]. Furthermore, investigations utilizing microglia-deficient AD mice by Kiani Shabestari et al. [76] proved that the hereditary microglia deficiency in AD models in mice results in a switch from parenchymal amyloid plaques to cerebral amyloid angiopathy, brain calcification and hemorrhages, and early death. Transplantation of adult microglia reverses these pathological alterations, demonstrating that microglia defend the brain from harmful co-pathologies associated withAD [76].

Aβ is toxic to neurons, with the oligomeric forms being more harmful than the fibrils [77]. For instance, in an *in vivo* investigation, only in the presence of amyloid aggregation does the injection of amyloid peptides into the dorsal dentate gyrus of rats cause spatial working memory deficit, synaptic dysfunction, cell death, and glial activation [78]. Meanwhile, the neurotoxicity of Aβ peptides is related to their aggregation propensity [79]. One possible factor related to oligomer toxicity is the exposed hydrophobic amino acid. As demonstrated in an *in vitro* study by Yoshiike et al. [80], exposed amino acids, in particular lysine and arginine, cause electrostatic and hydrophobic interactions with cells that may be reduced by covering or altering these amino acids. Another explanation is that the molecular shedding of oligomers from injected fibrils in model organisms may lead to fibrillar amyloid toxicity. Thus, the oligomer is an underlying driver of toxicity [81].

In many respects, Aβ is toxic to neurons. It may form ion-permeable pores, disturb intracellular calcium homeostasis, and induce membrane potential loss. It may also result in apoptosis, synapse loss, and cytoskeletal disruption [79]. Piling up studies indicates that, prior to neuronal death, synaptic dysfunction is vitally important in the initial phase of AD pathogenesis [82]. But how Aβ mediates its impacts on synaptic plasticity can take several years to figure out. For instance, in an *ex vivo* study, the protein interacting with C kinase 1 might help explain the impact of Aβ on synapses [83]. One possible cause of synaptic dysfunction in AD is glutamatergic neurotoxicity. Glutamate exerts its activity through ionotropic receptors (iGluRs) and metabotropic receptors (mGluRs). The iGluR family includes the N-methyl-d-aspartate receptor (NMDAR), the *α*-amino-3-hydroxy-5-methyl-4-isoxazolepropionic acid receptor (AMPAR), and the kainate receptor [84]. The effects of Aβ on NMDAR and AMPAR have been intensively investigated. For instance, long-term depression, spine shrinkage, and loss of synapses are all associated with AD and are driven by glutamate excitotoxicity as a consequence of Aβ-induced activation of the GluN2B NMDAR [85–87].

Both oligomeric Aβ and fibrillary Aβ have been shown to stimulate microglial synthesis as well as the release of pro-inflammatory cytokines such as IL1, IL6, and TNF*α*; chemokines including macrophage inflammatory protein-1 and monocyte chemotactic-1; free radicals such as reactive oxygen species, including superoxide anions and hydroxy radicals; complement components [77]. In addition, glutamate has been shown to contribute to microglia neurotoxicity in AD. The iGluRs and the mGluRs are both expressed in microglia, in which they play a significant role in the interaction between neurons and microglia and their activities. NMDARs, for example, are abundantly expressed on microglia, and their excessive activation can enhance Aβ and tau production. Furthermore, activation of microglial AMPARs contributes to the reduction in the pro-inflammatory cytokine TNF*α* as well as the upregulation of the anti-inflammatory cytokine IL-10 [84, 88]. The absence of microglial GluA2 (an AMPAR subunit), leads to the entry of Ca2 + into microglia in response to glutamate, and secretes pro-inflammatory cytokines, thereby increasing the toxicity of glutamate to neurons [84]. Regarding the mGluR family, seven mGluRs are expressed on microglia including mGluR 1-6 and 8 [84, 88]. Stimulating microglia via group-I and group-III mGluRs can result in a protective or detrimental phenotype [84, 89, 90]. For example, mGlu5 provides neuroprotection to inhibit the production of NO and TNF*α* and attenuate microglia-mediated neurotoxicity [84, 88]. Furthermore, the activation of Group-II mGluRs in microglia causes neurotoxic phenotypes, such as an increase in TNF*α* production, mitochondrial depolarization, and cell death. When Aβ stimulates microglia, glutamate is released and can generate autologous feedback that further activates microglia via the mGluRs of group II [84, 91, 92]. Meanwhile, the activation of group-II mGluRs leads to increased microglial Aβ uptake and clearance [84].

Aβ assemblies have various properties including monomers, oligomers, and fibrils. Microglia detect and bind to soluble Aβ oligomers, protofibrils, and insoluble fibrils through a variety of cell surface pattern recognition receptors, including the cell surface cluster of differentiation (CD) markers CD14, CD36, CD47, *α*6β1 integrin, class A1 scavenger receptor (SCARA1) and Toll-like receptors (TLRs). This binding leads to a transformation of resting microglia into activated cells, limiting plaque growth and accumulation. Internalization of Aβ-binding methoxy-X40 dye systemically injected at a higher rate near the plaque demonstrated the uptake of Aβ by microglia [73]. Because of microglial neuroprotective capability in Aβ clearance and degradation, as well as the production of antioxidants and neurotrophic factors, Aβ neurotoxicity is attenuated [77].

Therefore, early activation of microglia is beneficial since it eliminates Aβ plaques as well as dying or dead cells through phagocytosis [93]. Activated microglial phagocytosis of Aβ preventing plaque formation and deposition. However, chronic microglial activation may have harmful effects including the exacerbation of neuroinflammation, an increase of Aβ accumulation, and accentuation of neurodegeneration as a result of ineffectivephagocytosis.

## MICROGLIA AND TAU PATHOLOGY

Although the amyloid hypothesis is confirmed in AD, Aβ deposition is believed to be a required but insufficient prerequisite for the progression of AD [94]. The presence of tau has been shown to be necessary for Aβ toxicity [95, 96]. It is also worth noting that aggregation and spread of tau have been demonstrated to be significantly exacerbated by Aβ-induced microglial activation [97]. Activated microglia have been discovered to play a role in tau pathology either directly by causing neuroinflammation or indirectly by interfering with the homeostasis around the neurons [98]. Furthermore, the association between the quantity of activated microglia and the number of NFTs was stronger than the link between the activation of microglia and the distribution of amyloid plaques [99].

Microglia carry out a dual function in the pathology of tau. On the one hand, pathologically accumulated tau may be phagocytosed by microglia [100–103]. A study by Bolos et al. [101] reported that microglia colocalized with NFTs in postmortem brain tissue from AD patients. Aggregated tau was also internalized by these cells *in vivo* as well as *in vitro* [101]. In addition, microglia can internalize and degrade hyperphosphorylated tau that has been isolated from the brain tissue of postmortem AD patients or P301S transgenic mice’s brain tissue [100]. Aberrant activation of microglia, on the other hand, promotes tau pathology [104–107]. The CX3 C chemokine receptor 1 (CX3CR1) has been found to possess a key function in tau pathology mediated by microglia [104, 105]. The hippocampus and frontal cortices of AD brains were shown to have considerably lower levels of CX3 C chemokine ligand 1 (CX3CL1) and CX3CR1 compared to controls [105], suggesting that signaling through CX3CL1/CX3CR1 is impaired in AD. Moreover, CX3CR1-deficient AD transgenic mice displayed elevated tau phosphorylation [105]. Alterations in microglial activation have been consistently noted in tauopathy animals [104]. In animals with tauopathies, deletion of CX3CR1 results in an even greater elevation in microglial activation and phosphorylation of microtubule-associated protein tau. Furthermore, neurodegeneration induced by tau is also influenced by variations in genes expressed by microglia, such as colony-stimulating factor 1 receptor (*CSF1* *R*) [107–109], *APOE* [110, 111], and *TREM2* [112].

Aging is the primary risk factor for AD [113]. In this setting, microglia are thought to contribute to the development of the pathology by losing their neuroprotective capabilities, becoming more toxic, and altering how they respond to various stimuli, leading to the emergence of a senescent phenotype [114]. These age-related modifications in microglia have previously been identified which involve changes in cytokine releasee [115], increased expression of activation markers [116], and emergence of dystrophic morphologies [117]. It has been suggested that the removal of senescent microglia and their replacement by young microglia capable of performing the functions of the former may offer an effective treatment for AD [118]. To this end, it has been demonstrated that tau propagation and neurodegeneration can be blocked by pharmacologically depleting microglia [108, 119]. Further investigation also showed that glial cells from P301S mice contain senescent markers. According to this paradigm, the elimination of senescent cells inhibited gliosis, tau hyperphosphorylation, and neuronal degeneration, protecting cognitive function [120]. Similar to how it affected Aβ mouse models, this approach decreased the generation of senile plaques[75, 121].

The microglia not only internalize and degrade hyperphosphorylated tau but also participate in its spread [106, 107, 119]. Dujardin and Hyman [122] reported that tau proteins have been demonstrated to exhibit prion-like spreading abilities, either by active transmission from neuron to neuron or by infecting secondary cells via a seeding process. Microglia’s contribution to the spread of tau is still up for debate, though. In recent work, Wang et al. [123] looked at the function of microglial nuclear factor kappa-light-chain-enhancer of activated B cells (NF-*κ*B) signaling in tau processing, seeding, and spreading as well as tau toxicity using behavioral studies in conjunction with genetic deletion or activation of IkB kinase kinase in microglia. Their findings revealed that microglial NF-*κ*B activation contributes to disease progression intauopathy [123].

## ASTROCYTES IN THE CNS

Astrocytes, originating from neuroepithelium-derived radial glial cells [124], are the most numerous cell type in the brain comprising between 20% and 40% of all the cells. By the end of the 19th century, astrocytes have been already recognized as a morphologically heterogeneous population and classified as protoplasmic and fibrous based on their differences in cellular morphology and anatomical locations [125]. The substantial morphological variations between these two subpopulations of astrocytes were originally described using Golgi staining in combination with electron microscopy. This revealed that protoplasmic astrocytes are complicated cells with abundant fine processes that are localized in gray matter. Conversely, fibrous astrocytes are localized within the white matter, and they are less complex with little to moderate branching processes [126]. In addition to this classical morphological division, Emsley and Mackils [127] categorized astrocytes into nine subtypes by using three complementary methods for labeling astrocytes (transgenic hGFAP-GFP mice, GFAP immunostaining, and S100β immunostaining), including Bergmann glia, ependymal glia, “fibrous”, marginal glia, perivascular, “protoplasmic”, “radial”, tanycytes, and“velate”.

In a healthy brain, astrocytes are involved in multifaceted physiological functions determining the normal operation of the nervous tissue, including, but not limited to, modulating the brain microenvironment, maintaining blood-brain barrier integrity, supplying energy substrates to neurons, modulating synaptic activity, and maintaining fluid, ion, pH and neurotransmitter homeostasis [124]. Concurrently, astrocytes communicate with both neural and non-neural cells, including neurons and their synapses, microglia, oligodendrocytes, oligodendrocyte progenitor cells, circulating immune cells, meningeal fibroblasts and various perivascular cells [128].

Astrocytes are implicated in a wide variety of neurological disorders as they can guard the brain against damage and repair the neural tissue after the injury. Astrocytes become reactive in an injured condition or other pathological processes and converted into reactive astrogliosis. Reactive astrocytes undergo complex and conflicting region-specific alterations, including morphological, cellular, and functional changes compared to their normal counterparts [129]. Increased glial fibrillary acid protein (GFAP) expression is a feature of reactive astrocytes and is frequently used to identify the changes in astrocyte morphology such as hypertrophy [129].

In addition to classifying reactive astrocytes as proliferative broader-forming astrocytes and non-proliferative hypertrophic reactive astrocytes [128], the most well-known categorization of reactive astrocyte subtypes is that of the A1 (“pro-inflammatory”) and A2 (“anti-inflammatory”) phenotypes, which provide neurotoxic and neuroprotective effects, respectively [130] (see Fig. 1B). In a mouse experiment, specific cytokines secreted by microglia exposed to LPS caused A1 astrocytes to lose many of their normal astrocytic functions such as promoting neuronal survival and outgrowth, and significantly upregulate several classical complement cascade genes that have been previously reported as being destructive to synapses. Additionally, they secrete neurotoxins that rapidly kill neurons and mature differentiated oligodendrocytes [130]. In contrast, ischemic stroke-induced A2 astrocytes upregulate neurotrophic or anti-inflammatory genes that promote neuronal survival and tissue repair [130]. The recently published consensus statement, however, emphasizes the gaps in the use of these binary divisions of reactive astrocytes, for instance, A1-versus-A2, good-versus-bad, or neurotoxic-versus-neuroprotective. Furthermore, the authors argue for the promotion of reactive astrocyte research with the evaluation of multiple molecular and functional parameters in conjunction with multivariate statistical methods and the determination of the impact on pathological hallmarks [131]. Moreover, A1 astrocytes are not always harmful such as the deletion of A1 astrocytes in a murine prion disease model results in accelerated neurodegenerative disease progression [132]. Thus, the A1/A2 dichotomy is challenged and the effect of reactive astrogliosis is complicated.

## THE DUAL ROLE OF ASTROCYTES IN AD

Emerging lines of evidence have confirmed that massive reactive astrogliosis is an archetypical morphological feature in the brain of AD mouse models [133] and AD patients [134]. In AD, astrocytes experience remodeling in morphology, transcriptional profile, and function. Morphologically, they may become either atrophy or hypertrophy. Astrocytes located away from the amyloid deposits undergo atrophy, while astrocytes surrounding the plaques develop hypertrophy. Atrophic reactive astrocytes are found in the CA1 hippocampal region, dentate gyrus, entorhinal cortex, and medial prefrontal cortex in the 3xTg-AD [135–137] and PDAPP-J20 mice [138]. In these mouse models, morphological atrophy of astrocytes occurs even before the emergence of amyloid plaques. Hypertrophic reactive astrocytes are found to accumulate around amyloid plaques with a dense layer of processes as if forming a scar-like physical barrier around them, perhaps acting as neuroprotective barriers [126]. As the disease progresses, the number of reactive astrocytes in close proximity to amyloid plaques increases and is independent of plaque size and the apolipoprotein E (*APOE*) gene [139]. A study conducted by Diaz-Amarilla et al. [140] reported that conditioned media from aged astrocytes (3xTg-AD) that were transgenic for AD displayed neurotoxic effects *in vitro*. As opposed to media derived from young astrocytes [140]. It was discovered that activation of glycogen synthase kinase-3, a kinase that participates in the hyperphosphorylation of tau, is required for these effects, which also resulted in a proinflammatory response. Similar to how IP3 receptor type 2 expression in astrocytes and decreased calcium signaling in astrocytes in human brains from AD patients, in the APPNL-F mouse model for AD [141], these changes were related to the early changes in functional connectivity and network activity. As astrocytes were restored to normal calcium signaling, neuronal hyperactivity, seizure susceptibility, behavioral disturbances, and aberrant functional connections were all addressed.

Unlike microglia, where Aβ itself may be the key chemotactic signal for them, astrocytes might mainly respond to plaque-associated neuritic damage, as shown in AD postmortem human tissue [142]. A major question for astrocytes in AD is whether they are innocent bystanders or pivotal players in the progression of the disease. A plethora of studies have displayed astrocyte involvement in the clearance of Aβ *in vitro*, indicating their role in attenuating neurodegenerative processes in AD [143–145]. For example, it has been demonstrated that astrocytes can internalize Aβ_1 - 42_ in mice [145] and human cells [146, 147] as well as internalize Aβ plaques through enzymatic cleavage [148]. Neprilysin [149] and insulin-degrading enzyme [150] are expressed at higher levels in AD mice, which helps the astrocytes remove Aβ, whereas the elimination of extracellular Aβ is promoted by astrocyte-derived matrix metalloproteinase (MMP)-2 and MMP-9 [148]. Meanwhile, AD conditions may affect astrocytes turning them into Aβ producers. In support, TGF-β1 [151] alone or interferon-*γ* (IFN-*γ*) in combination with TNF*α* [152, 153] or IL1β [152] can drive astrocytes to produce Aβ. In addition, astrocytes may engulf large amounts of partly digested Aβ protofibrils, ultimately resulting in the reduction of astrocytic degradation capacity and apoptosis of neurons, as shown in *in vitro* and *in vivo* studies [154].

*APOE*, the most powerful genetic risk factor for AD [155], has three primary isoforms, *APOE* E2, *APOE* E3, and *APOE* E4, with *APOE* E3 being the most widely expressed [156]. Comparatively, to noncarriers, *APOE* E4 carriers have impaired memory function, more rapid cognitive decline, and exacerbated AD pathology [157]. ApoE is principally produced in a subset of astrocytes in the CNS, where it functions as a secreted lipid-transport protein that transports lipids between organs [158, 159]. It is unclear how exactly ApoE variations affect AD pathogenesis, although it is likely to be related to Aβ accumulation and clearance in the brain [155]. Studies in AD transgenic mice have revealed that *APOE* E4 has a pathogenic role in the development of AD by impairing astrocyte activation, which leads to synaptic loss, as well as by gaining harmful activities after interacting with Aβ [160]. Furthermore, compared with individuals with the *APOE* E3 or other isoforms, human carriers of the *APOE* E4 allele exhibit higher levels of Aβ plaques [161]. Interestingly, *APOE* E4 ablation specifically in astrocytes reduces tau that has been found to be phosphorylated and associated with tau’s neurodegeneration [162]. The *APOE* E4 allele and the pathogenesis of AD are connected by these findings, implying that *APOE* E4, which is generated from astrocytes, could be a factor in regulating tauopathies [155].

Even while AD mostly exhibits neuronal tau pathology, thorn-shaped astrocytes with perinuclear tau deposits have been observed [163, 164], especially in models of aging-related tau astrogliopathy [165]. Additionally, the correlation between aberrant aggregates of tau on astrocytes and neurodegeneration suggests that astrocytes have the ability to internalize this protein [166]. As astrocytes show enrichment for proteostatic, inflammatory, and metal ion homeostasis pathways, transcriptome analysis of postmortem brains from individuals with AD revealed changes in glial gene expression were linked to levels of amyloid or phosphorylated tau in the tissue [167]. In fact, risk loci related to tauopathy mediated by astrocytes, which include genes for clustering, myocyte enhancer factor 2 C, and IQ domain-containing protein K, have been discovered in postmortem brains of AD patients using single-nuclei RNA-sequencing transcriptomics [167].

The role of astrocytes in the evolution of NFTs in AD has attracted far less attention. However, studies have demonstrated reactive astrocytes can penetrate the extracellular ghost NFTs with their processes in advanced AD [168]. Thus, these NFTs may display both tau and GFAP immunoreactivities [169, 170]. A further finding from postmortem research is that the quantity of reactive astrocytes is correlated with the number of tangles and the stage of NFTs formation in the para-hippocampal cortex [171]. Collectively, these findings indicate that astrocytes participate in NFT progressions in AD.

## CROSSTALK BETWEEN MICROGLIA AND ASTROCYTES

Major types of glial cells in the brain include microglia, astrocytes, and oligodendrocytes, and the ratio of glia to neurons in the brains of humans and other primates is closer to 1 : 1 [172]. Glia crosstalk is pivotal for brain development, function, and disease. There is a constant fine and intimate crosstalk between microglia and astrocytes, thus influencing one another’s activity. The molecular conversation between them is maintained in part via secreted mediators, such as cytokines, chemokines, growth factors, mitogenic factors, NO, reactive oxygen species, neurotransmitters, gliotransmitters, innate immune mediators, tissue damage molecules such as adenosine triphosphate (ATP), and metabolic mediators such as glutamate, that may involve in cellular metabolism and mediate tissue changes [173]. Additionally, communication among microglia, astrocytes, and neurons is through extracellular vesicles (EVs) release and response. Exosomes and microvesicles are examples of EVs that act as cell communicators and immune response regulators. They may also function as biomarkers for diseases and as components of medicine delivery systems. Due to their ability to be secreted and absorbed by both cell types, EVs are crucial mediators of communication between microglia and astrocytes [174, 175]. EVs, with the capability of transporting cargo packaged by the originating cells, may engage in the pathogenesis of neurodegenerative disorders through the transport and transfer of toxic aggregates, such as tau and Aβ in AD [176]. In a rodent model of AD, for example, microglia were shown to spread tau via exosome secretion and depletion of microglia dramatically reduced tau propagation [177]. Besides, a recent study described the importance of microglia in Aβ phagocytosis and the propagation of Aβ pathology by invading non-diseased brain tissues [178]. Moreover, an *in vitro* study showed that microglia internalize exosomes released from cells overexpressing AβPP. In addition to the ability of exosomes to induce microglial activation and release of proinflammatory cytokines [179]. Furthermore, it has been reported that activated microglia-derived exosomes spread the inflammatory milieu composed of proteins implicated in cellular adhesion/extracellular matrix organization, cellular metabolism, and autophagy-lysosomal pathway, which modulate astrocyte activity [180].

**Fig. 2:**
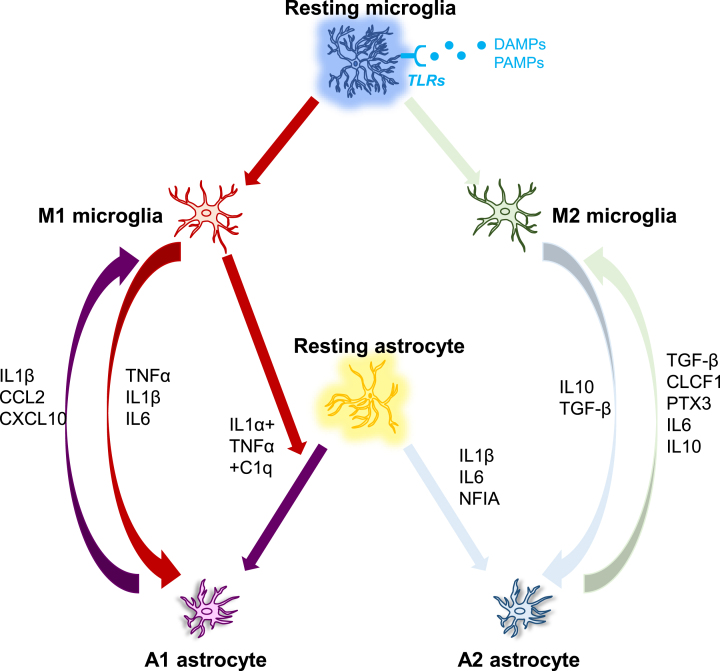
**The interplay between microglia and astrocytes.** DAMP/PAMP signaling activates microglia via TLR receptors, which then regulate the phenotypes of astrocytes, which can range from neurotoxic to neuroprotective. Microglia and astrocytes can have a direct effect on each other via numerous molecules as demonstrated in Fig. 2. C1q, Complement component 1q; CCL2, C-C motif ligand 2; CLCF1, Cardiotrophin-like cytokine factor 1; CXCL10, C-X-C, motif chemokine ligand 10; IFN-*γ*, Interferon gamma; IL, Interleukin; NFIA, Nuclear factor IA; PTX3, Pentraxin 3; TGF-β, Transforming growth factor-beta 1; TNF*α*, Tumor necrosis factor alpha.

In diverse neuropathologies, microglia are activated earlier than astrocytes. For instance, in cultured human fetal microglia and astrocytes, astrocytes responded to the microglia-secreted product IL1β, but not the primary stimulus LPS [181], indicating that astrocyte activation may be a secondary consequence of microglial activation. In AD, pattern recognition receptors such as TLRs are considered to be implicated in triggering glial activation. To date, ten human TLRs and thirteen murine TLRs have been described, although TLR10 is non-functional in mice [182]. They can recognize both external pathogen-associated molecular patterns (PAMPs) and internal DAMPs. Numerous cytokines and chemokines derived from activated microglia cause an immune response when DAMPs or PAMPs are detected [183]. Human microglia express TLR1-13 except for TLR10 and mouse microglia express TLR1-10 [184], while astrocytes express TLR1-5 and TLR9 in humans as well as TLR1-9 in mice [185]. Probably because astrocytes express comparatively low levels of TLRs, they cannot directly build up responses to pathogens but require the presence of microglia to sense the pathogen and secrete signals to trigger their activation [186]. The lack of response from human astrocytes to LPS stimulation may be explained by the low levels of TLR4 expression in these cells, which are important for detecting LPS from Gram-negative bacteria [186]. These results together imply that microglia appear to be more susceptible to pathogen recognition than astrocytes, and the probable pattern of glial activation is that microglia become activated to develop an innate immune response upon entry of a pathogen into the CNS, activated microglia then send inflammatory cytokine-mediated activation signals to reactive astrocytes [186] (see Fig. 2).

The β-chemokines are major chemoattractant molecules that affect cell motility, and in addition to their activation, microglia and astrocytes must migrate to the site of the injury. Apart from the variations in their chemokine secretion, both glia exhibit distinct motile responses. Human microglia are stimulated to migrate when CCL2, CCL3, and CCL4 are introduced to chemotaxis chambers, but astrocytes are not [187]. However, one conflicting research reports that CCR2, the main CCL2 receptor that is mainly detected in mouse and human microglia [188], has been identified in cultured human fetal astrocytes and acted as a mediator in their chemotaxis [189].

Astrocytes release a variety of chemokines including CCL2, CXCL1 (GRO-*α*), CXCL10 (IP-10), and CXCL12 (SDF-1), as well as microglia expressing certain matching chemokine receptors, such as CCL2, CXCL12, etc., implying a strong relation may exist between microglia and astrocytes. Changes to chemokine levels may be relevant to AD pathological changes. CCL2 level is correlated with impaired memory [190, 191], and this fact has been evidenced in the plasma of MCI and AD patients with higher CCL2 [192, 193]. Moreover, CCL4 is expressed widely in reactive astrocytes in AD brains, as revealed by immunohistochemical staining [194]. Although it is unknown if CCL4 has any other effects, the absence of a chemotactic response in human astrocytes shows that astrocytes can employ CCL4 to signal to microglia. Human microglia and astrocytes can both express and secret IL1β and TNF*α* [195, 196], which suggests that both cells are able to induce chemotaxis of other glial cells through the exchange of activation signals.

The appropriate interrelationship between microglia and astrocytes in the course of the disease has a significant impact on astrocytes, supporting neuronal function and survival after acute injury. On the other hand, dysregulated microglia-astrocyte interactions can result in neuroinflammation in AD. As proinflammatory stimuli cause brain disorders, microglia act as the first line of defense. Activated M2 microglia produce anti-inflammatory properties IL10 that communicates with IL10 receptor (IL10 R), which is mostly expressed in A2 astrocytes. This causes the astrocyte to secrete TGF-β. TGF-β is a neuroprotective molecule that works to reduce inflammation while supporting the M2 noninflammatory phenotype of microglia [197]. TGF-β has also been found to protect synapses against the deleterious effect of Aβ oligomers in the AD model [198]. Additionally, impaired TGF-β signaling is observed in the AD brain [199, 200]. However, transmitting an inflammatory message to astrocytes can sometimes have an adverse effect on the CNS environment, leading to excessive activation that can cause neurodegeneration instead of protecting it [186]. For instance, cytokines released from activated microglia and comprised of IL1*α*, TNF*α*, and complement factor C1q, polarize astrocytes toward a neurotoxic phenotype (A1). These A1 astrocytes lose many normal functions, including phagocytosis and promoting neuronal survival [186]. TNF*α* serving as a critical driver of astrocyte activation has also been demonstrated in human-induced pluripotent stem cell-derived astrocytes [201]. A1 reactive astrocytes were also detected in aged brains, but the aging-induced upregulation reactive genes by astrocytes were reduced in knockout mice that genetically ablated the three microglial factors known to induce astrocytic polarization of A1, including TNF*α* [202]. For most patients affected by neurodegenerative disorders, reactive astrocytes are ubiquitous in the tissue of the CNS [203]. Hence, the interaction of activated microglia with astrocytes is crucial to the neuroinflammatory process. These inflammatory signals may be amplified, and it is possible that neuroinflammation from different neurological illnesses like AD uses similar molecular languages to trigger reactivity astrocytes [204].

Studies on AD conducted both *in vitro* and *in vivo* have shown that impaired astrocytic activation alters the nature of microglia. For example, in APP/PS1 GFAP^–/–^vim^–/–^ mice, poor astrocyte activation increased the abundance of microglia around plaques, which may be a compensatory mechanism for resolving the ineffective role of astrocytes as proposed by the study [205]. Since, astrocytes modulate microglial activation, as described by the study [206], conditioned media obtained from Aβ-induced microglia-astrocyte coculture rather than the Aβ-induced microglia culture lose their neurotoxic potential in hippocampus culture. In addition, Gfa2-VIVIT-mediated suppression of the astrocytic calcineurin/nuclear factor of activated T cells signaling pathway resulted in improved cognition, ameliorated synaptic dysfunction, attenuated glial activation, and diminished Aβ pathology in APP/PS1 mice [207].

**Fig. 3:**
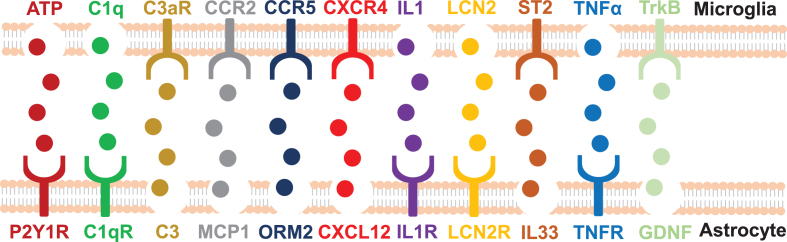
**Molecular conversation between microglia and astrocytes.** The signaling pathway between microglia and astrocytes is listed here briefly (modified from Li et al. [215]). ATP, Adenosine triphosphate; C1q, Complement component 1q; C1qR, C1q: Complement component 1q receptor; C3aR, C3a receptor; CCR, C-C chemokine receptor; CXCL12, C-X-C motif chemokine ligand 12; CXCR4, C-X-C chemokine receptor type 4; GDNF, Glial cell line-derived neurotrophic factor; IL, Interleukin; LCN2, Lipocalin 2; MCP1, Monocyte chemoattractant protein 1; ORM2, Orosomucoid 2; P2Y, metabotropic; ST2, Suppressor of tumorigenicity 2; TNF, Tumor necrosis factor; TrkB, Tropomyosin-related kinase B.

The bidirectional communication between microglia and astrocytes may extend to numerous small molecules released by astrocytes (see Fig. 3). For example, neuron-generated Aβ activates NF-*κ*B signaling in astrocytes, releasing complement C3 as a result. Afterward, complement C3 binds with the C3a receptor (C3aR) on microglial and neurons to disrupt cognitive function and impede Aβ phagocytosis. More astrocytes and microglia become activated in response to damaged neurons. The pathogenic cycle is primarily promoted by complement-dependent intercellular crosstalk, and the feedforward loop can be successfully blocked by utilizing a C3aR antagonist. Thus, the astrocytic C3/C3a-microglial C3aR axis has proven to govern Aβ dynamics and AD neuropathology [200, 208]. Another concrete example is the endogenous protein orosomucoid-2 (ORM2) which is predominantly expressed and produced by astrocytes during inflammation. At the same time, astrocytic ORM2 modulates microglial migration and activation through blockage of microglial C–C chemokine receptor type 5, resulting in an anti-inflammatory outcome [172, 209]. Besides, it was noted that plasminogen activator inhibitor type 1 (PAI-1), a physiological inhibitor of tissue type and urokinase-type plasminogen activators (tPA and uPA), function as a major mediator of microglia-to-astrocyte crosstalk. PAI-1 protein levels have been reported to be increased in the plasma and brain tissues of patients with AD [210]. In animal studies, the deletion of PAI-1 markedly reduced cerebral Aβ burden in APP/PS1 mice [211]. Likewise, TM5275, an orally bioavailable small molecule PAI-1 inhibitor, reduced hippocampal and cortical Aβ load as well as improved learning/memory function in double APP/PS1 transgenic mice [212]. In the CNS, astrocytes are the major cellular source of PAI-1. Both microglia and astrocytes have been shown to secrete PAI-1 under inflammatory conditions [172]. PAI-1 is known to promote microglial migration through the low-density lipoprotein receptor-related protein (LRP)-1/Janus kinase (JAK)/STAT1 axis. Similarly, PAI-1 modulates the phagocytic activity of microglial cells in a vitronectin- and Toll-like receptor 2/6-dependent manner, indicating that PAI-1 derived from glia (mainly astrocytes) acts as a regulator in the migration and phagocytosis of microglia in an paracrine or autocrine manner [172]. Moreover, glial cell line-released cerebral BDNF, dopamine neurotrophic factor, and neurotrophic factor (GDNF), released from glial cell lines are some of the important astrocyte-derived molecules involved in modulating microglial activation. Recent studies determined that astrocytic GDNF can control the activation of microglia in the midbrain, thereby controlling or slowing neurodegenerative progression by inhibiting neuroinflammation [213, 214]. Taken together, these findings point out a tight correlation between microglia and astrocytes. Furthermore, these molecular mediators involved in the crosstalk between microglia and crosstalk open up new therapeutic opportunities for the treatment of AD.

## CONCLUSION

Studies of the intercommunication between microglia and astrocytes in recent years have offered novel and meaningful insights into the CNS in both health and disease. In normal brain physiology, it is noticeable that the conversation between microglia and astrocytes takes place via secreted molecules, and it is possible that molecular alterations in their interaction may underlie or promote disease states. Both microglia and astrocytes have the ability to control each other’s fate and are actively involved in the close reciprocal regulation of CNS insult and injury. Typically, microglial cells respond early to pathological insults in the brain, followed by astrocytic reactions. Both glial cells release different signaling molecules to establish their mutual communication or to give autoregulatory feedback. Importantly, astrocytes exhibit a dual function in neuroinflammatory disorders, not only can enhance immune responses and postpone restoration but also can limit neuroinflammation and become neuroprotective. The scientific understanding of this bidirectional communication between microglia and astrocytes is changing dramatically, and it is greatly improving our understanding of neuroscience. Current therapeutic approaches for AD and clinical trials in the foundation for suppressing inflammatory immune response are lacking. The microglia-astrocyte dialogue sheds light on important aspects of this intricate system, which is made up of several unidentified functional cells and cells with an unfathomable range of diversity and flexibility. With advances in technology, microglia-astrocyte communication will become an effective and accurate target for future AD treatment.

## References

[1] (2021) 2021 Alzheimer’s disease facts and figures. Alzheimers Dement 17, 327–406. 33756057 10.1002/alz.12328

[2] Zhou R , Ji B , Kong Y , Qin L , Ren W , Guan Y , Ni R (2021) PET imaging of neuroinflammation in Alzheimer’s disease. Front Immunol 12, 739130. 34603323 10.3389/fimmu.2021.739130 PMC8481830

[3] Glenner GG , Wong CW (1984) Alzheimer’s disease: Initial report of the purification and characterization of a novel cerebrovascular amyloid protein. Biochem Biophys Res Commun 120, 885–890. 6375662 10.1016/s0006-291x(84)80190-4

[4] Masters CL , Simms G , Weinman NA , Multhaup G , McDonald BL , Beyreuther K (1985) Amyloid plaque core protein in Alzheimer disease and Down syndrome. Proc Natl Acad Sci U S A 82, 4245–4249. 3159021 10.1073/pnas.82.12.4245 PMC397973

[5] Wischik C , Novak M , Edwards P , Klug A , Tichelaar W , Crowther R (1988) Structural characterization of the core of the paired helical filament of Alzheimer disease. Proc Natl Acad Sci U S A 85, 4884–4888. 2455299 10.1073/pnas.85.13.4884 PMC280541

[6] Braak E , Griffing K , Arai K , Bohl J , Bratzke H , Braak H (1999) Neuropathology of Alzheimer’s disease: What is new since A. Alzheimer? Eur Arch Psychiatry Clin Neurosci 249, S14–S22. 10.1007/pl00014168 10654095

[7] Mann DM , Yates PO , Marcyniuk B (1985) Correlation between senile plaque and neurofibrillary tangle counts in cerebral cortex and neuronal counts in cortex and subcortical structures in Alzheimer’s disease. Neurosci Lett 56, 51–55. 4011048 10.1016/0304-3940(85)90439-2

[8] Mann DM (1985) The neuropathology of Alzheimer’s disease: A review with pathogenetic, aetiological and therapeutic considerations. Mech Ageing Dev 31, 213–255. 3906293 10.1016/0047-6374(85)90092-2

[9] Rama Rao KV , Kielian T (2015) Neuron–astrocyte interactions in neurodegenerative diseases: Role of neuroinflammation. Clin Exp Neuroimmunol 6, 245–263. 26543505 10.1111/cen3.12237 PMC4629520

[10] Terry RD , Davies P (1980) Dementia of the Alzheimer type. Annu Rev Neurosci 3, 77–95. 6251745 10.1146/annurev.ne.03.030180.000453

[11] Fakhoury M (2018) Microglia and astrocytes in Alzheimer’s disease: Implications for therapy. Curr Neuropharmacol 16, 508–518. 28730967 10.2174/1570159X15666170720095240 PMC5997862

[12] Kaur D , Sharma V , Deshmukh R (2019) Activation of microglia and astrocytes: A roadway to neuroinflammation and Alzheimer’s disease. Inflammopharmacology 27, 663–677. 30874945 10.1007/s10787-019-00580-x

[13] Kwon HS , Koh S-H (2020) Neuroinflammation in neurodegenerative disorders: The roles of microglia and astrocytes. Transl Neurodegener 9, 42. 33239064 10.1186/s40035-020-00221-2 PMC7689983

[14] Dá Mesquita S , Ferreira AC , Sousa JC , Correia-Neves M , Sousa N , Marques F (2016) Insights on the pathophysiology of Alzheimer’s disease: The crosstalk between amyloid pathology, neuroinflammation and the peripheral immune system. Neurosci Biobehav Rev 68, 547–562. 27328788 10.1016/j.neubiorev.2016.06.014

[15] Lai KSP , Liu CS , Rau A , Lanctôt KL , Köhler CA , Pakosh M , Carvalho AF , Herrmann N (2017) Peripheral inflammatory markers in Alzheimer’s disease: A systematic review and meta-analysis of 175 studies. J Neurol Neurosurg Psychiatry 88, 876–882. 28794151 10.1136/jnnp-2017-316201

[16] Tao Q , Ang TFA , DeCarli C , Auerbach SH , Devine S , Stein TD , Zhang X , Massaro J , Au R , Qiu WQ (2018) Association of chronic low-grade inflammation with risk of Alzheimer disease in ApoE4 carriers. JAMA Network Open 1, e183597. 30646251 10.1001/jamanetworkopen.2018.3597 PMC6324596

[17] La Rosa F , Saresella M , Baglio F , Piancone F , Marventano I , Calabrese E , Nemni R , Ripamonti E , Cabinio M , Clerici M (2017) Immune and imaging correlates of mild cognitive impairment conversion to Alzheimer’s disease. Sci Rep 7, 16760. 29196629 10.1038/s41598-017-16754-y PMC5711836

[18] Wendeln A-C , Degenhardt K , Kaurani L , Gertig M , Ulas T , Jain G , Wagner J , Häsler LM , Wild K , Skodras A (2018) Innate immune memory in the brain shapes neurological disease hallmarks. Nature 556, 332–338. 29643512 10.1038/s41586-018-0023-4 PMC6038912

[19] Zenaro E , Pietronigro E , Bianca VD , Piacentino G , Marongiu L , Budui S , Turano E , Rossi B , Angiari S , Dusi S (2015) Neutrophils promote Alzheimer’s disease–like pathology and cognitive decline via LFA-1 integrin. Nat Med 21, 880–886. 26214837 10.1038/nm.3913

[20] Tejera D , Mercan D , Sanchez-Caro JM , Hanan M , Greenberg D , Soreq H , Latz E , Golenbock D , Heneka MT (2019) Systemic inflammation impairs microglial Aβ clearance through NLRP 3 inflammasome. EMBO J 38, e101064. 31359456 10.15252/embj.2018101064 PMC6717897

[21] Baruch K , Rosenzweig N , Kertser A , Deczkowska A , Sharif AM , Spinrad A , Tsitsou-Kampeli A , Sarel A , Cahalon L , Schwartz M (2015) Breaking immune tolerance by targeting Foxp3+regulatory T cells mitigates Alzheimer’s disease pathology. Nat Commun 6, 7967. 26284939 10.1038/ncomms8967 PMC4557123

[22] Ortí-Casañ N , Zuhorn IS , Naudé PJ , De Deyn PP , van Schaik PE , Wajant H , Eisel UL (2022) A TNF receptor 2 agonist ameliorates neuropathology and improves cognition in an Alzheimer’s disease mouse model. Proc Natl Acad Sci U S A 119, e2201137119. 36037389 10.1073/pnas.2201137119 PMC9482428

[23] Jung S , Schwartz M (2012) Non-identical twins–microglia and monocyte-derived macrophages in acute injury and autoimmune inflammation. Front Immunol 3, 89. 22566968 10.3389/fimmu.2012.00089 PMC3345364

[24] Moreira EG , Boll KM , Correia DG , Soares JF , Rigobello C , Maes M (2019) Why should psychiatrists and neuroscientists worry about paraoxonase 1? Curr Neuropharmacol 17, 1004–1020. 30592255 10.2174/1570159X17666181227164947 PMC7052826

[25] Zotova E , Holmes C , Johnston D , Neal JW , Nicoll JA , Boche D (2011) Microglial alterations in human Alzheimer’s disease following Aβ42 immunization. Neuropathol Appl Neurobiol 37, 513–524. 21166690 10.1111/j.1365-2990.2010.01156.x

[26] Block ML , Zecca L , Hong J-S (2007) Microglia-mediated neurotoxicity: Uncovering the molecular mechanisms. Nat Rev Neurosci 8, 57–69. 17180163 10.1038/nrn2038

[27] Lawson LJ , Perry VH , Dri P , Gordon S (1990) Heterogeneity in the distribution and morphology of microglia in the normal adult mouse brain. Neuroscience 39, 151–170. 2089275 10.1016/0306-4522(90)90229-w

[28] Ginhoux F , Greter M , Leboeuf M , Nandi S , See P , Gokhan S , Mehler MF , Conway SJ , Ng LG , Stanley ER (2010) Fate mapping analysis reveals that adult microglia derive from primitive macrophages. Science 330, 841–845. 20966214 10.1126/science.1194637 PMC3719181

[29] Alliot F , Lecain E , Grima B , Pessac B (1991) Microglial progenitors with a high proliferative potential in the embryonic and adult mouse brain. Proc Natl Acad Sci U S A 88, 1541–1545. 1996355 10.1073/pnas.88.4.1541 PMC51055

[30] Alliot F , Godin I , Pessac B (1999) Microglia derive from progenitors, originating from the yolk sac, and which proliferate in the brain. Dev Brain Res 117, 145–152. 10567732 10.1016/s0165-3806(99)00113-3

[31] Hristovska I , Pascual O (2016) Deciphering resting microglial morphology and process motility from a synaptic prospect. Front Integr Neurosci 9, 73. 26834588 10.3389/fnint.2015.00073 PMC4717304

[32] Kettenmann H , Kirchhoff F , Verkhratsky A (2013) Microglia: New roles for the synaptic stripper. Neuron 77, 10–18. 23312512 10.1016/j.neuron.2012.12.023

[33] Nimmerjahn A , Kirchhoff F , Helmchen F (2005) Resting microglial cells are highly dynamic surveillants of brain parenchyma *in vivo*. Science 308, 1314–1318. 15831717 10.1126/science.1110647

[34] Davalos D , Grutzendler J , Yang G , Kim JV , Zuo Y , Jung S , Littman DR , Dustin ML , Gan W-B (2005) ATP mediates rapid microglial response to local brain injury *in vivo*. Nat Neurosci 8, 752–758. 15895084 10.1038/nn1472

[35] Roth TL , Nayak D , Atanasijevic T , Koretsky AP , Latour LL , McGavern DB (2014) Transcranial amelioration of inflammation and cell death after brain injury. Nature 505, 223–228. 24317693 10.1038/nature12808 PMC3930079

[36] Town T , Nikolic V , Tan J (2005) The microglial “activation” continuum: From innate to adaptive responses. J Neuroinflammation 2, 24. 16259628 10.1186/1742-2094-2-24 PMC1298325

[37] Sierra A , Beccari S , Diaz-Aparicio I , Encinas JM , Comeau S , Tremblay M-È (2014) Surveillance, phagocytosis, and inflammation: How never-resting microglia influence adult hippocampal neurogenesis. Neural Plast 2014, 610343. 24772353 10.1155/2014/610343 PMC3977558

[38] Otxoa-de-Amezaga A , Miró-Mur F , Pedragosa J , Gallizioli M , Justicia C , Gaja-Capdevila N , Ruíz-Jaen F , Salas-Perdomo A , Bosch A , Calvo M (2019) Microglial cell loss after ischemic stroke favors brain neutrophil accumulation. Acta Neuropathol 137, 321–341. 30580383 10.1007/s00401-018-1954-4 PMC6513908

[39] Boche D , Perry V , Nicoll J (2013) Activation patterns of microglia and their identification in the human brain. Neuropathol Appl Neurobiol 39, 3–18. 23252647 10.1111/nan.12011

[40] Saijo K , Glass CK (2011) Microglial cell origin and phenotypes in health and disease. Nat Rev Immunol 11, 775–787. 22025055 10.1038/nri3086

[41] Varnum MM , Ikezu TJAiete (2012) The classification of microglial activation phenotypes on neurodegeneration and regeneration in Alzheimer’s disease brain. Arch Immunol Ther Exp (Warsz) 60, 251–266. 22710659 10.1007/s00005-012-0181-2 PMC4429536

[42] Hu X , Leak RK , Shi Y , Suenaga J , Gao Y , Zheng P , Chen J (2015) Microglial and macrophage polarization—new prospects for brain repair. Nat Rev Neurol 11, 56–64. 25385337 10.1038/nrneurol.2014.207 PMC4395497

[43] Kigerl KA , Gensel JC , Ankeny DP , Alexander JK , Donnelly DJ , Popovich PG (2009) Identification of two distinct macrophage subsets with divergent effects causing either neurotoxicity or regeneration in the injured mouse spinal cord. J Neurosci 29, 13435–13444. 19864556 10.1523/JNEUROSCI.3257-09.2009 PMC2788152

[44] Perego C , Fumagalli S , De Simoni M-G (2011) Temporal pattern of expression and colocalization of microglia/macrophage phenotype markers following brain ischemic injury in mice. J Neuroinflammation 8, 1–20. 22152337 10.1186/1742-2094-8-174 PMC3251548

[45] Wang G , Zhang J , Hu X , Zhang L , Mao L , Jiang X , Liou AK-F , Leak RK , Gao Y , Chen J (2013) Microglia/macrophage polarization dynamics in white matter after traumatic brain injury. J Cereb Blood Flow Metab 33, 1864–1874. 23942366 10.1038/jcbfm.2013.146 PMC3851898

[46] Hu X , Li P , Guo Y , Wang H , Leak RK , Chen S , Gao Y , Chen J (2012) Microglia/macrophage polarization dynamics reveal novel mechanism of injury expansion after focal cerebral ischemia. Stroke 43, 3063–3070. 22933588 10.1161/STROKEAHA.112.659656

[47] Morganti JM , Riparip L-K , Rosi S (2016) Call off the dog (ma): M1/M2 polarization is concurrent following traumatic brain injury. PLoS One 11, e0148001. 26808663 10.1371/journal.pone.0148001 PMC4726527

[48] Cherry JD , Olschowka JA , O’Banion MK (2014) Neuroinflammation and M2 microglia: The good, the bad, and the inflamed. J Neuroinflammation 11, 98. 24889886 10.1186/1742-2094-11-98 PMC4060849

[49] Chakrabarty P , Tianbai L , Herring A , Ceballos-Diaz C , Das P , Golde TE (2012) Hippocampal expression of murine IL-4 results in exacerbation of amyloid deposition. Mol Neurodegener 7, 36. 22838967 10.1186/1750-1326-7-36 PMC3441281

[50] Kiyota T , Okuyama S , Swan RJ , Jacobsen MT , Gendelman HE , Ikezu T (2010) CNS expression of anti-inflammatory cytokine interleukin-4 attenuates Alzheimer’s disease-like pathogenesis in APP+PS1 bigenic mice. FASEB J 24, 3093. 20371618 10.1096/fj.10-155317 PMC2909296

[51] Keren-Shaul H , Spinrad A , Weiner A , Matcovitch-Natan O , Dvir-Szternfeld R , Ulland TK , David E , Baruch K , Lara-Astaiso D , Toth B (2017) A unique microglia type associated with restricting development of Alzheimer’s disease. Cell 169, 1276–1290. e1217. 28602351 10.1016/j.cell.2017.05.018

[52] Wang H (2021) Microglia heterogeneity in Alzheimer’s disease: Insights from single-cell technologies. Front Synaptic Neurosci 13, 68. 10.3389/fnsyn.2021.773590 PMC8735255 35002670

[53] Hansen DV , Hanson JE , Sheng M (2018) Microglia in Alzheimer’s disease. J Cell Biol 217, 459–472. 29196460 10.1083/jcb.201709069 PMC5800817

[54] Friedman BA , Srinivasan K , Ayalon G , Meilandt WJ , Lin H , Huntley MA , Cao Y , Lee S-H , Haddick PC , Ngu H (2018) Diverse brain myeloid expression profiles reveal distinct microglial activation states and aspects of Alzheimer’s disease not evident in mouse models. Cell Rep 22, 832–847. 29346778 10.1016/j.celrep.2017.12.066

[55] Deczkowska A , Keren-Shaul H , Weiner A , Colonna M , Schwartz M , Amit I (2018) Disease-associated microglia: A universal immune sensor of neurodegeneration. Cell 173, 1073–1081. 29775591 10.1016/j.cell.2018.05.003

[56] Gabandé-Rodríguez E , Keane L , Capasso M (2020) Microglial phagocytosis in aging and Alzheimer’s disease. J Neurosci Res 98, 284–298. 30942936 10.1002/jnr.24419

[57] Amor S , Puentes F , Baker D , Van Der Valk P (2010) Inflammation in neurodegenerative diseases. Immunology 129, 154–169. 20561356 10.1111/j.1365-2567.2009.03225.x PMC2814458

[58] Kohman RA , Rhodes JS (2013) Neurogenesis, inflammation and behavior. Brain Behav Immun 27, 22–32. 22985767 10.1016/j.bbi.2012.09.003 PMC3518576

[59] Hein AM , O’Banion MK (2009) Neuroinflammation and memory: The role of prostaglandins. Mol Neurobiol 40, 15–32. 19365736 10.1007/s12035-009-8066-z PMC3124778

[60] Rao JS , Kellom M , Kim H-W , Rapoport SI , Reese EA (2012) Neuroinflammation and synaptic loss. Neurochem Res 37, 903–910. 22311128 10.1007/s11064-012-0708-2 PMC3478877

[61] Wisniewski H , Barcikowska M , Kida E (1991) Phagocytosis of β/A4 amyloid fibrils of the neuritic neocortical plaques. Acta Neuropathol 81, 588–590. 1858487 10.1007/BF00310142

[62] Wisniewski H , Wegiel J , Wang K , Kujawa M , Lach B (1989) Ultrastructural studies of the cells forming amyloid fibers in classical plaques. Can J Neurol Sci 16, 535–542. 2553231 10.1017/s0317167100029887

[63] Akiyama H , Mori H , Saido T , Kondo H , Ikeda K , McGeer PL (1999) Occurrence of the diffuse amyloid β-protein (Aβ) deposits with numerous Aβ-containing glial cells in the cerebral cortex of patients with Alzheimer’s disease. Glia 25, 324–331. 10028915 10.1002/(sici)1098-1136(19990215)25:4<324::aid-glia2>3.0.co;2-5

[64] Mackenzie IR , Hao C , Munoz DG (1995) Role of microglia in senile plaque formation. Neurobiol Aging 16, 797–804. 8532113 10.1016/0197-4580(95)00092-s

[65] Dickson D , Farlo J , Davies P , Crystal H , Fuld P , Yen S-H (1988) Alzheimer’s disease. A double-labeling immunohistochemical study of senile plaques. Am J Pathol 132, 86. 2456021 PMC1880629

[66] Haga S , Akai K , Ishii T (1989) Demonstration of microglial cells in and around senile (neuritic) plaques in the Alzheimer brain: An immunohistochemical study using a novel monoclonal antibody. Acta Neuropathol 77, 569–575. 2750476 10.1007/BF00687883

[67] Malm TM , Koistinaho M , Pärepalo M , Vatanen T , Ooka A , Karlsson S , Koistinaho J (2005) Bone-marrow-derived cells contribute to the recruitment of microglial cells in response to β-amyloid deposition in APP/PS1 double transgenic Alzheimer mice. Neurobiol Dis 18, 134–142. 15649704 10.1016/j.nbd.2004.09.009

[68] Wegiel J , Wang K-C , Imaki H , Rubenstein R , Wronska A , Osuchowski M , Lipinski WJ , Walker LC , LeVine H (2001) The role of microglial cells and astrocytes in fibrillar plaque evolution in transgenic APPsw mice. Neurobiol Aging 22, 49–61. 11164276 10.1016/s0197-4580(00)00181-0

[69] Wegiel J , Imaki H , Wang K-C , Wegiel J , Wronska A , Osuchowski M , Rubenstein R (2003) Origin and turnover of microglial cells in fibrillar plaques of APPsw transgenic mice. Acta Neuropathol 105, 393–402. 12624793 10.1007/s00401-002-0660-3

[70] Wegiel J , Imaki H , Wang K-C , Wegiel J , Rubenstein R (2004) Cells of monocyte/microglial lineage are involved in both microvessel amyloidosis and fibrillar plaque formation in APPsw tg mice. Brain Res 1022, 19–29. 15353209 10.1016/j.brainres.2004.06.058

[71] Simon E , Obst J , Gomez-Nicola D (2019) The evolving dialogue of microglia and neurons in Alzheimer’s disease: Microglia as necessary transducers of pathology. Neuroscience 405, 24–34. 29427657 10.1016/j.neuroscience.2018.01.059

[72] Bacskai BJ , Hyman BT (2002) Alzheimer’s disease: What multiphoton microscopy teaches us. Neuroscientist 8, 386–390. 12374422 10.1177/107385802236963

[73] Bolmont T , Haiss F , Eicke D , Radde R , Mathis CA , Klunk WE , Kohsaka S , Jucker M , Calhoun ME (2008) Dynamics of the microglial/amyloid interaction indicate a role in plaque maintenance. J Neurosci 28, 4283–4292. 18417708 10.1523/JNEUROSCI.4814-07.2008 PMC3844768

[74] Casali BT , MacPherson KP , Reed-Geaghan EG , Landreth GE (2020) Microglia depletion rapidly and reversibly alters amyloid pathology by modification of plaque compaction and morphologies. Neurobiol Dis 142, 104956. 32479996 10.1016/j.nbd.2020.104956 PMC7526856

[75] Spangenberg E , Severson PL , Hohsfield LA , Crapser J , Zhang J , Burton EA , Zhang Y , Spevak W , Lin J , Phan NY (2019) Sustained microglial depletion with CSF1R inhibitor impairs parenchymal plaque development in an Alzheimer’s disease model. Nat Commun 10, 3758. 31434879 10.1038/s41467-019-11674-z PMC6704256

[76] Shabestari SK , Morabito S , Danhash EP , McQuade A , Sanchez JR , Miyoshi E , Chadarevian JP , Claes C , Coburn MA , Hasselmann J (2022) Absence of microglia promotes diverse pathologies and early lethality in Alzheimer’s disease mice. Cell Rep 39, 110961. 35705056 10.1016/j.celrep.2022.110961 PMC9285116

[77] Mizuno T (2012) The biphasic role of microglia in Alzheimer’s disease. Int J Alzheimers Dis 2012, 737846. 22655214 10.1155/2012/737846 PMC3357927

[78] Stephan A , Laroche S , Davis S (2001) Generation of aggregated β-amyloid in the rat hippocampus impairs synaptic transmission and plasticity and causes memory deficits. J Neurosci 21, 5703–5714. 11466442 10.1523/JNEUROSCI.21-15-05703.2001 PMC6762634

[79] Reiss AB , Arain HA , Stecker MM , Siegart NM , Kasselman LJ (2018) Amyloid toxicity in Alzheimer’s disease. Rev Neurosci 29, 613–627. 29447116 10.1515/revneuro-2017-0063

[80] Yoshiike Y , Akagi T , Takashima A (2007) Surface structure of amyloid-β fibrils contributes to cytotoxicity. Biochemistry 46, 9805–9812. 17676931 10.1021/bi700455c

[81] Tipping KW , Karamanos TK , Jakhria T , Iadanza MG , Goodchild SC , Tuma R , Ranson NA , Hewitt EW , Radford SE (2015) pH-induced molecular shedding drives the formation of amyloid fibril-derived oligomers. Proc Natl Acad Sci U S A 112, 5691–5696. 25902516 10.1073/pnas.1423174112 PMC4426459

[82] Anggono V , Tsai L-H , Götz J (2016) Glutamate receptors in Alzheimer’s disease: Mechanisms and therapies. Neural Plast 2016, 8256196. 27293907 10.1155/2016/8256196 PMC4884857

[83] Alfonso S , Kessels HW , Banos CC , Chan TR , Lin ET , Kumaravel G , Scannevin RH , Rhodes KJ , Huganir R , Guckian KM (2014) Synapto-depressive effects of amyloid beta require PICK 1. Eur J Neurosci 39, 1225–1233. 24713001 10.1111/ejn.12499 PMC3983572

[84] Zhang X , Wang D , Zhang B , Zhu J , Zhou Z , Cui L (2020) Regulation of microglia by glutamate and its signal pathway in neurodegenerative diseases. Drug Discov Today 25, 1074–1085. 32320851 10.1016/j.drudis.2020.04.001

[85] Kullmann DM , Lamsa KP (2007) Long-term synaptic plasticity in hippocampal interneurons. Nat Rev Neurosci 8, 687–699. 17704811 10.1038/nrn2207

[86] Babaei P (2021) NMDA and AMPA receptors dysregulation in Alzheimer’s disease. Eur J Pharmacol 908, 174310. 34265291 10.1016/j.ejphar.2021.174310

[87] Liu J , Chang L , Song Y , Li H , Wu Y (2019) The role of NMDA receptors in Alzheimer’s disease. Front Neurosci 13, 43. 30800052 10.3389/fnins.2019.00043 PMC6375899

[88] Stolero N , Frenkel D (2021) The dialog between neurons and microglia in Alzheimer’s disease: The neurotransmitters view. J Neurochem 158, 1412–1424. 33314073 10.1111/jnc.15262

[89] Taylor DL , Diemel LT , Pocock JM (2003) Activation of microglial group III metabotropic glutamate receptors protects neurons against microglial neurotoxicity. J Neurosci 23, 2150–2160. 12657674 10.1523/JNEUROSCI.23-06-02150.2003 PMC6742009

[90] Williams CJ , Dexter DT (2014) Neuroprotective and symptomatic effects of targeting group III mG lu receptors in neurodegenerative disease. J Neurochem 129, 4–20. 24224472 10.1111/jnc.12608

[91] Taylor D , Diemel L , Cuzner M , Pocock J (2002) Activation of group II metabotropic glutamate receptors underlies microglial reactivity and neurotoxicity following stimulation with chromogranin A, a peptide up-regulated in Alzheimer’s disease. J Neurochem 82, 1179–1191. 12358765 10.1046/j.1471-4159.2002.01062.x

[92] Taylor DL , Jones F , Kubota ESCS , Pocock JM (2005) Stimulation of microglial metabotropic glutamate receptor mGlu2 triggers tumor necrosis factor *α*-induced neurotoxicity in concert with microglial-derived Fas ligand. J Neurosci 25, 2952–2964. 15772355 10.1523/JNEUROSCI.4456-04.2005 PMC6725132

[93] Hickman SE , Allison EK , El Khoury J (2008) Microglial dysfunction and defective β-amyloid clearance pathways in aging Alzheimer’s disease mice. J Neurosci 28, 8354–8360. 18701698 10.1523/JNEUROSCI.0616-08.2008 PMC2597474

[94] Perea JR , Bolós M , Avila J (2020) Microglia in Alzheimer’s disease in the context of tau pathology. Biomolecules 10, 1439. 33066368 10.3390/biom10101439 PMC7602223

[95] Rapoport M , Dawson HN , Binder LI , Vitek MP , Ferreira A (2002) Tau is essential to β-amyloid-induced neurotoxicity. Proc Natl Acad Sci U S A 99, 6364–6369. 11959919 10.1073/pnas.092136199 PMC122954

[96] Roberson ED , Scearce-Levie K , Palop JJ , Yan F , Cheng IH , Wu T , Gerstein H , Yu G-Q , Mucke L (2007) Reducing endogenous tau ameliorates amyloid ß-induced deficits in an Alzheimer’s disease mouse model. Science 316, 750–754. 17478722 10.1126/science.1141736

[97] Stancu I-C , Cremers N , Vanrusselt H , Couturier J , Vanoosthuyse A , Kessels S , Lodder C , Brône B , Huaux F , Octave J-N (2019) Aggregated tau activates NLRP3–ASC inflammasome exacerbating exogenously seeded and non-exogenously seeded tau pathology *in vivo*. Acta Neuropathol 137, 599–617. 30721409 10.1007/s00401-018-01957-y PMC6426830

[98] Song L , Wells EA , Robinson AS (2021) Critical molecular and cellular contributors to tau pathology. Biomedicines 9, 190. 33672982 10.3390/biomedicines9020190 PMC7918468

[99] Serrano-Pozo A , Mielke ML , Gómez-Isla T , Betensky RA , Growdon JH , Frosch MP , Hyman BT (2011) Reactive glia not only associates with plaques but also parallels tangles in Alzheimer’s disease. Am J Pathol 179, 1373–1384. 21777559 10.1016/j.ajpath.2011.05.047 PMC3157187

[100] Luo W , Liu W , Hu X , Hanna M , Caravaca A , Paul SM (2015) Microglial internalization and degradation of pathological tau is enhanced by an anti-tau monoclonal antibody. Sci Rep 5, 11161. 26057852 10.1038/srep11161 PMC4460904

[101] Bolós M , Llorens-Martín M , Jurado-Arjona J , Hernández F , Rábano A , Avila J (2016) Direct evidence of internalization of tau by microglia *in vitro* and *in vivo*. J Alzheimers Dis 50, 77–87. 26638867 10.3233/JAD-150704

[102] Brelstaff J , Tolkovsky AM , Ghetti B , Goedert M , Spillantini MG (2018) Living neurons with tau filaments aberrantly expose phosphatidylserine and are phagocytosed by microglia. Cell Rep 24, 1939–1948.e1934. 30134156 10.1016/j.celrep.2018.07.072 PMC6161320

[103] Hopp SC , Lin Y , Oakley D , Roe AD , DeVos SL , Hanlon D , Hyman BT (2018) The role of microglia in processing and spreading of bioactive tau seeds in Alzheimer’s disease. J Neuroinflammation 15, 1–15. 30227881 10.1186/s12974-018-1309-z PMC6145371

[104] Bhaskar K , Konerth M , Kokiko-Cochran ON , Cardona A , Ransohoff RM , Lamb BT (2010) Regulation of tau pathology by the microglial fractalkine receptor. Neuron 68, 19–31. 20920788 10.1016/j.neuron.2010.08.023 PMC2950825

[105] Cho S-H , Sun B , Zhou Y , Kauppinen TM , Halabisky B , Wes P , Ransohoff RM , Gan L (2011) CX3CR1 protein signaling modulates microglial activation and protects against plaque-independent cognitive deficits in a mouse model of Alzheimer disease. J Biol Chem 286, 32713–32722. 21771791 10.1074/jbc.M111.254268 PMC3173153

[106] Maphis N , Xu G , Kokiko-Cochran ON , Jiang S , Cardona A , Ransohoff RM , Lamb BT , Bhaskar K (2015) Reactive microglia drive tau pathology and contribute to the spreading of pathological tau in the brain. Brain 138, 1738–1755. 25833819 10.1093/brain/awv081 PMC4542622

[107] Clayton K , Delpech JC , Herron S , Iwahara N , Ericsson M , Saito T , Saido TC , Ikezu S , Ikezu T (2021) Plaque associated microglia hyper-secrete extracellular vesicles and accelerate tau propagation in a humanized APP mouse model. Mol Neurodegener 16, 1–16. 33752701 10.1186/s13024-021-00440-9 PMC7986521

[108] Mancuso R , Fryatt G , Cleal M , Obst J , Pipi E , Monzón-Sandoval J , Ribe E , Winchester L , Webber C , Nevado A (2019) CSF1R inhibitor JNJ-40346527 attenuates microglial proliferation and neurodegeneration in P301S mice. Brain 142, 3243–3264. 31504240 10.1093/brain/awz241 PMC6794948

[109] Johnson NR , Yuan P , Castillo E , Lopez TP , Yue W , Bond A , Rivera BM , Sullivan MC , Hirouchi M , Giles K (2023) CSF1R inhibitors induce a sex-specific resilient microglial phenotype and functional rescue in a tauopathy mouse model. Nat Commun 14, 118. 36624100 10.1038/s41467-022-35753-w PMC9829908

[110] Shi Y , Yamada K , Liddelow SA , Smith ST , Zhao L , Luo W , Tsai RM , Spina S , Grinberg LT , Rojas JC (2017) ApoE4 markedly exacerbates tau-mediated neurodegeneration in a mouse model of tauopathy. Nature 549, 523–527. 28959956 10.1038/nature24016 PMC5641217

[111] Shi Y , Manis M , Long J , Wang K , Sullivan PM , Remolina Serrano J , Hoyle R , Holtzman DM (2019) Microglia drive APOE-dependent neurodegeneration in a tauopathy mouse model. J Exp Med 216, 2546–2561. 31601677 10.1084/jem.20190980 PMC6829593

[112] Leyns CE , Gratuze M , Narasimhan S , Jain N , Koscal LJ , Jiang H , Manis M , Colonna M , Lee VM , Ulrich JD (2019) TREM2 function impedes tau seeding in neuritic plaques. Nat Neurosci 22, 1217–1222. 31235932 10.1038/s41593-019-0433-0 PMC6660358

[113] Barnes DE , Yaffe K (2011) The projected effect of risk factor reduction on Alzheimer’s disease prevalence. Lancet Neurol 10, 819–828. 21775213 10.1016/S1474-4422(11)70072-2 PMC3647614

[114] Luo X-G , Ding J-Q , Chen S-D (2010) Microglia in the aging brain: Relevance to neurodegeneration. Mol Neurodegener 5, 12. 20334662 10.1186/1750-1326-5-12 PMC2852379

[115] Sierra A , Gottfried-Blackmore AC , McEwen BS , Bulloch K (2007) Microglia derived from aging mice exhibit an altered inflammatory profile. Glia 55, 412–424. 17203473 10.1002/glia.20468

[116] Perry V , Matyszak M , Fearn S (1993) Altered antigen expression of microglia in the aged rodent CNS. Glia 7, 60–67. 8423063 10.1002/glia.440070111

[117] Streit WJ , Sammons NW , Kuhns AJ , Sparks DL (2004) Dystrophic microglia in the aging human brain. Glia 45, 208–212. 14730714 10.1002/glia.10319

[118] Han J , Zhu K , Zhang XM , Harris RA (2019) Enforced microglial depletion and repopulation as a promising strategy for the treatment of neurological disorders. Glia 67, 217–231. 30378163 10.1002/glia.23529 PMC6635749

[119] Asai H , Ikezu S , Tsunoda S , Medalla M , Luebke J , Haydar T , Wolozin B , Butovsky O , Kügler S , Ikezu T (2015) Depletion of microglia and inhibition of exosome synthesis halt tau propagation. Nat Neurosci 18, 1584–1593. 26436904 10.1038/nn.4132 PMC4694577

[120] Bussian TJ , Aziz A , Meyer CF , Swenson BL , van Deursen JM , Baker DJ (2018) Clearance of senescent glial cells prevents tau-dependent pathology and cognitive decline. Nature 562, 578–582. 30232451 10.1038/s41586-018-0543-y PMC6206507

[121] Sosna J , Philipp S , Albay R , Reyes-Ruiz JM , Baglietto-Vargas D , LaFerla FM , Glabe CG (2018) Early long-term administration of the CSF1R inhibitor PLX3397 ablates microglia and reduces accumulation of intraneuronal amyloid, neuritic plaque deposition and pre-fibrillar oligomers in 5XFAD mouse model of Alzheimer’s disease. Mol Neurodegener 13, 1–11. 29490706 10.1186/s13024-018-0244-x PMC5831225

[122] Dujardin S , Hyman BT (2020) Tau prion-like propagation: State of the art and current challenges. Adv Exp Med Biol 1184, 305–325. 10.1007/978-981-32-9358-8_23 32096046

[123] Wang C , Fan L , Khawaja RR , Liu B , Zhan L , Kodama L , Chin M , Li Y , Le D , Zhou Y (2022) Microglial NF-*κ*B drives tau spreading and toxicity in a mouse model of tauopathy. Nat Commun 13, 1969. 35413950 10.1038/s41467-022-29552-6 PMC9005658

[124] Verkhratsky A , Nedergaard M (2018) Physiology of astroglia. Physiol Rev 98, 239–389. 29351512 10.1152/physrev.00042.2016 PMC6050349

[125] Ramón y Cajal S (1909) Histologie du système nerveux de l’homme & des vertébrés. Maloine, Paris.

[126] Sofroniew MV , Vinters HV (2010) Astrocytes: Biology and pathology. Acta Neuropathol 119, 7–35. 20012068 10.1007/s00401-009-0619-8 PMC2799634

[127] Emsley JG , Macklis JD (2006) Astroglial heterogeneity closely reflects the neuronal-defined anatomy of the adult murine CNS. Neuron Glia Biol 2, 175–186. 17356684 10.1017/S1740925X06000202 PMC1820889

[128] Sofroniew MV (2020) Astrocyte reactivity: Subtypes, states, and functions in CNS innate immunity. Trends Immunol 41, 758–770. 32819810 10.1016/j.it.2020.07.004 PMC7484257

[129] Pekny M , Pekna M (2014) Astrocyte reactivity and reactive astrogliosis: Costs and benefits. Physiol Rev 94, 1077–1098. 25287860 10.1152/physrev.00041.2013

[130] Liddelow SA , Guttenplan KA , Clarke LE , Bennett FC , Bohlen CJ , Schirmer L , Bennett ML , Münch AE , Chung W-S , Peterson TC (2017) Neurotoxic reactive astrocytes are induced by activated microglia. Nature 541, 481–487. 28099414 10.1038/nature21029 PMC5404890

[131] Escartin C , Galea E , Lakatos A , O’Callaghan JP , Petzold GC , Serrano-Pozo A , Steinhäuser C , Volterra A , Carmignoto G , Agarwal A (2021) Reactive astrocyte nomenclature, definitions, and future directions. Nat Neurosci 24, 312–325. 33589835 10.1038/s41593-020-00783-4 PMC8007081

[132] Hartmann K , Sepulveda-Falla D , Rose IV , Madore C , Muth C , Matschke J , Butovsky O , Liddelow S , Glatzel M , Krasemann S (2019) Complement 3+-astrocytes are highly abundant in prion diseases, but their abolishment led to an accelerated disease course and early dysregulation of microglia. Acta Neuropathol Commun 7, 83. 31118110 10.1186/s40478-019-0735-1 PMC6530067

[133] Matsuoka Y , Picciano M , Malester B , LaFrancois J , Zehr C , Daeschner JM , Olschowka JA , Fonseca MI , O’Banion MK , Tenner AJ (2001) Inflammatory responses to amyloidosis in a transgenic mouse model of Alzheimer’s disease. Am J Pathol 158, 1345–1354. 11290552 10.1016/S0002-9440(10)64085-0 PMC1891893

[134] Nagele RG , D’Andrea MR , Lee H , Venkataraman V , Wang H-Y (2003) Astrocytes accumulate Aβ42 and give rise to astrocytic amyloid plaques in Alzheimer disease brains. Brain Res 971, 197–209. 12706236 10.1016/s0006-8993(03)02361-8

[135] Kulijewicz-Nawrot M , Verkhratsky A , Chvatal A , Sykova E , Rodríguez JJ (2012) Astrocytic cytoskeletal atrophy in themedial prefrontal cortex of a triple transgenic mouse model ofAlzheimer’s disease. J Anat 221, 252–262. 22738374 10.1111/j.1469-7580.2012.01536.x PMC3458630

[136] Olabarria M , Noristani HN , Verkhratsky A , Rodríguez JJ (2010) Concomitant astroglial atrophy and astrogliosis in a triple transgenic animal model of Alzheimer’s disease. Glia 58, 831–838. 20140958 10.1002/glia.20967

[137] Yeh C-Y , Vadhwana B , Verkhratsky A , Rodríguez JJ (2011) Early astrocytic atrophy in the entorhinal cortex of a triple transgenic animal model of Alzheimer’s disease. ASN Neuro 3, AN20110025. 10.1042/AN20110025 PMC3243908 22103264

[138] Beauquis J , Pavía P , Pomilio C , Vinuesa A , Podlutskaya N , Galvan V , Saravia F (2013) Environmental enrichment prevents astroglial pathological changes in the hippocampus of APP transgenic mice, model of Alzheimer’s disease. Exp Neurol 239, 28–37. 23022919 10.1016/j.expneurol.2012.09.009

[139] Perez-Nievas BG , Serrano-Pozo A (2018) Deciphering the astrocyte reaction in Alzheimer’s disease. Front Aging Neurosci 10, 114. 29922147 10.3389/fnagi.2018.00114 PMC5996928

[140] Diaz-Amarilla P , Arredondo F , Dapueto R , Boix V , Carvalho D , Santi MD , Vasilskis E , Mesquita-Ribeiro R , Dajas-Bailador F , Abin-Carriquiry JA (2022) Isolation and characterization of neurotoxic astrocytes derived from adult triple transgenic Alzheimer’s disease mice. Neurochem Int 159, 105403. 35853553 10.1016/j.neuint.2022.105403

[141] Shah D , Gsell W , Wahis J , Luckett ES , Jamoulle T , Vermaercke B , Preman P , Moechars D , Hendrickx V , Jaspers T (2022) Astrocyte calcium dysfunction causes early network hyperactivity in Alzheimer’s disease. Cell Rep 40, 111280. 36001964 10.1016/j.celrep.2022.111280 PMC9433881

[142] Simpson J , Ince P , Lace G , Forster G , Shaw P , Matthews F , Savva G , Brayne C , Wharton S (2010) Astrocyte phenotype in relation to Alzheimer-type pathology in the ageing brain. Neurobiol Aging 31, 578–590. 18586353 10.1016/j.neurobiolaging.2008.05.015

[143] Koistinaho M , Lin S , Wu X , Esterman M , Koger D , Hanson J , Higgs R , Liu F , Malkani S , Bales KR (2004) Apolipoprotein E promotes astrocyte colocalization and degradation of deposited amyloid-β peptides. Nat Med 10, 719–726. 15195085 10.1038/nm1058

[144] Liu R-X , Huang C , Bennett DA , Li H , Wang R (2016) The characteristics of astrocyte on Aβ clearance altered in Alzheimer’s disease were reversed by anti-inflammatory agent (+)-2-(1-hydroxyl-4-oxocyclohexyl) ethyl caffeate. Am J Transl Res 8, 4082. 27829994 PMC5095303

[145] Wyss-Coray T , Loike JD , Brionne TC , Lu E , Anankov R , Yan F , Silverstein SC , Husemann J (2003) Adult mouse astrocytes degrade amyloid-β *in vitro* and in situ. Nat Med 9, 453–457. 12612547 10.1038/nm838

[146] Montoliu-Gaya L , Mulder SD , Veerhuis R , Villegas S (2017) Effects of an Aβ-antibody fragment on Aβ aggregation and astrocytic uptake are modulated by apolipoprotein E and J mimetic peptides. PLoS One 12, e0188191. 29155887 10.1371/journal.pone.0188191 PMC5695774

[147] Liu C-C , Hu J , Zhao N , Wang J , Wang N , Cirrito JR , Kanekiyo T , Holtzman DM , Bu G (2017) Astrocytic LRP1 mediates brain Aβ clearance and impacts amyloid deposition. J Neurosci 37, 4023–4031. 28275161 10.1523/JNEUROSCI.3442-16.2017 PMC5391682

[148] Yin K-J , Cirrito JR , Yan P , Hu X , Xiao Q , Pan X , Bateman R , Song H , Hsu F-F , Turk J (2006) Matrix metalloproteinases expressed by astrocytes mediate extracellular amyloid-β peptide catabolism. J Neurosci 26, 10939–10948. 17065436 10.1523/JNEUROSCI.2085-06.2006 PMC6674654

[149] Apelt J , Ach K , Schliebs R (2003) Aging-related down-regulation of neprilysin, a putative β-amyloid-degrading enzyme, in transgenic Tg2576 Alzheimer-like mouse brain is accompanied by an astroglial upregulation in the vicinity of β-amyloid plaques. Neurosci Lett 339, 183–186. 12633883 10.1016/s0304-3940(03)00030-2

[150] Leal MC , Dorfman VB , Gamba AF , Frangione B , Wisniewski T , Castaño EM , Sigurdsson EM , Morelli L (2006) Plaque-associated overexpression of insulin-degrading enzyme in the cerebral cortex of aged transgenic tg2576 mice with Alzheimer pathology. J Neuropathol Exp Neurol 65, 976–987. 17021402 10.1097/01.jnen.0000235853.70092.ba

[151] Lesné S , Docagne F , Gabriel C , Liot G , Lahiri DK , Buée L , Plawinski L , Delacourte A , MacKenzie ET , Buisson A (2003) Transforming growth factor-β1 potentiates amyloid-β generation in astrocytes and in transgenic mice. J Biol Chem 278, 18408–18418. 12626500 10.1074/jbc.M300819200

[152] Blasko I , Veerhuis R , Stampfer-Kountchev M , Saurwein-Teissl M , Eikelenboom P , Grubeck-Loebenstein B (2000) Costimulatory effects of interferon-*γ* and interleukin-1β or tumor necrosis factor *α* on the synthesis of Aβ1-40 and Aβ1-42 by human astrocytes. Neurobiol Dis 7, 682–689. 11114266 10.1006/nbdi.2000.0321

[153] Zhao J , O’Connor T , Vassar R (2011) The contribution of activated astrocytes to Aβ production: Implications for Alzheimer’s disease pathogenesis. J Neuroinflammation 8, 1–17. 22047170 10.1186/1742-2094-8-150 PMC3216000

[154] Söllvander S , Nikitidou E , Brolin R , Söderberg L , Sehlin D , Lannfelt L , Erlandsson A (2016) Accumulation of amyloid-β by astrocytes result in enlarged endosomes and microvesicle-induced apoptosis of neurons. Mol Neurodegener 11, 1–19. 27176225 10.1186/s13024-016-0098-z PMC4865996

[155] Wang P , Ye Y (2021) Astrocytes in neurodegenerative diseases: A perspective from tauopathy and *α*-synucleinopathy. Life 11, 938. 34575087 10.3390/life11090938 PMC8471224

[156] Huang Y , Mahley RW (2014) Apolipoprotein E: Structure and function in lipid metabolism, neurobiology, and Alzheimer’s diseases. Neurobiol Dis 72, 3–12. 25173806 10.1016/j.nbd.2014.08.025 PMC4253862

[157] Liu C-C , Kanekiyo T , Xu H , Bu G (2013) Apolipoprotein E and Alzheimer disease: Risk, mechanisms and therapy. Nat Rev Neurol 9, 106–118. 23296339 10.1038/nrneurol.2012.263 PMC3726719

[158] Pitas RE , Boyles JK , Lee SH , Foss D , Mahley RW (1987) Astrocytes synthesize apolipoprotein E and metabolize apolipoprotein E-containing lipoproteins. Biochim Biophys Acta 917, 148–161. 3539206 10.1016/0005-2760(87)90295-5

[159] Xu Q , Bernardo A , Walker D , Kanegawa T , Mahley RW , Huang Y (2006) Profile and regulation of apolipoprotein E (ApoE) expression in the CNS in mice with targeting of green fluorescent protein gene to the ApoE locus. J Neurosci 26, 4985–4994. 16687490 10.1523/JNEUROSCI.5476-05.2006 PMC6674234

[160] Wisniewski T , Drummond E (2020) APOE-amyloid interaction: Therapeutic targets. Neurobiol Dis 138, 104784. 32027932 10.1016/j.nbd.2020.104784 PMC7118587

[161] Carter DB (2005) The interaction of amyloid-beta with ApoE. Subcell Biochem 38, 255–272. 15709483 10.1007/0-387-23226-5_13

[162] Wang C , Xiong M , Gratuze M , Bao X , Shi Y , Andhey PS , Manis M , Schroeder C , Yin Z , Madore C (2021) Selective removal of astrocytic APOE4 strongly protects against tau-mediated neurodegeneration and decreases synaptic phagocytosis by microglia. Neuron 109, 1657–1674.e1657. 33831349 10.1016/j.neuron.2021.03.024 PMC8141024

[163] Kahlson MA , Colodner KJ (2015) Glial tau pathology in tauopathies: Functional consequences. J Exp Neurosci 9(Suppl 2), 43–50. 10.4137/JEN.S25515 PMC4750898 26884683

[164] Ferrer I , López-González I , Carmona M , Arregui L , Dalfó E , Torrejón-Escribano B , Diehl R , Kovacs GG (2014) Glial and neuronal tau pathology in tauopathies: Characterization of disease-specific phenotypes and tau pathology progression. J Neuropathol Exp Neurol 73, 81–97. 24335532 10.1097/NEN.0000000000000030

[165] Leyns CE , Holtzman DM (2017) Glial contributions to neurodegeneration in tauopathies. Mol Neurodegener 12, 50. 28662669 10.1186/s13024-017-0192-x PMC5492997

[166] Amro Z , Yool AJ , Collins-Praino LE (2021) The potential role of glial cells in driving the prion-like transcellular propagation of tau in tauopathies. Brain Behav Immun Health 14, 100242. 34589757 10.1016/j.bbih.2021.100242 PMC8474563

[167] Smith AM , Davey K , Tsartsalis S , Khozoie C , Fancy N , Tang SS , Liaptsi E , Weinert M , McGarry A , Muirhead RC (2022) Diverse human astrocyte and microglial transcriptional responses to Alzheimer’s pathology. Acta Neuropathol 143, 75–91. 34767070 10.1007/s00401-021-02372-6 PMC8732962

[168] Ikeda K , Haga C , Akiyama H , Kase K , Iritani S (1992) Coexistence of paired helical filaments and glial filaments in astrocytic processes within ghost tangles. Neurosci Lett 148, 126–128. 1338646 10.1016/0304-3940(92)90820-w

[169] Irwin DJ , Cohen TJ , Grossman M , Arnold SE , Xie SX , Lee VM-Y , Trojanowski JQ (2012) Acetylated tau, a novel pathological signature in Alzheimer’s disease and other tauopathies. Brain 135, 807–818. 22366796 10.1093/brain/aws013 PMC3286338

[170] Probst A , Ulrich J , Heitz PU (1982) Senile dementia of Alzheimer type: Astroglial reaction to extracellular neurofibrillary tangles in the hippocampus. Acta Neuropathol 57, 75–79. 7090745 10.1007/BF00688880

[171] Sheng JG , Mrak RE , Griffin WST (1997) Glial-neuronal interactions in Alzheimer disease: Progressive association of IL-1*α*+microglia and S100β+astrocytes with neurofibrillary tangle stages. J Neuropathol Exp Neurol 56, 285–290. 9056542

[172] Jha MK , Jo M , Kim J-H , Suk K (2019) Microglia-astrocyte crosstalk: An intimate molecular conversation. Neuroscientist 25, 227–240. 29931997 10.1177/1073858418783959

[173] Matejuk A , Ransohoff RM (2020) Crosstalk between astrocytes and microglia: An overview. Front Immunol 11, 1416. 32765501 10.3389/fimmu.2020.01416 PMC7378357

[174] Dickens AM , Tovar-y-Romo LB , Yoo S-W , Trout AL , Bae M , Kanmogne M , Megra B , Williams DW , Witwer KW , Gacias M (2017) Astrocyte-shed extracellular vesicles regulate the peripheral leukocyte response to inflammatory brain lesions. Sci Signal 10, eaai7696. 28377412 10.1126/scisignal.aai7696 PMC5590230

[175] Budnik V , Ruiz-Cañada C , Wendler F (2016) Extracellular vesicles round off communication in the nervous system. Nat Rev Neurosci 17, 160–172. 26891626 10.1038/nrn.2015.29 PMC4989863

[176] Paolicelli RC , Bergamini G , Rajendran L (2019) Cell-to-cell communication by extracellular vesicles: Focus on microglia. Neuroscience 405, 148–157. 29660443 10.1016/j.neuroscience.2018.04.003

[177] Goetzl EJ , Kapogiannis D , Schwartz JB , Lobach IV , Goetzl L , Abner EL , Jicha GA , Karydas AM , Boxer A , Miller BL (2016) Decreased synaptic proteins in neuronal exosomes of frontotemporal dementia and Alzheimer’s disease. FASEB J 30, 4141–4148. 27601437 10.1096/fj.201600816R PMC5102122

[178] d’Errico P , Ziegler-Waldkirch S , Aires V , Hoffmann P , Mezö C , Erny D , Monasor LS , Liebscher S , Ravi VM , Joseph K (2022) Microglia contribute to the propagation of Aβ into unaffected brain tissue. Nat Neurosci 25, 20–25. 34811521 10.1038/s41593-021-00951-0 PMC8737330

[179] Fernandes A , Ribeiro AR , Monteiro M , Garcia G , Vaz AR , Brites D (2018) Secretome from SH-SY5Y APPSwe cells trigger time-dependent CHME3 microglia activation phenotypes, ultimately leading to miR-21 exosome shuttling. Biochimie 155, 67–82. 29857185 10.1016/j.biochi.2018.05.015

[180] Drago F , Lombardi M , Prada I , Gabrielli M , Joshi P , Cojoc D , Franck J , Fournier I , Vizioli J , Verderio C (2017) ATP modifies the proteome of extracellular vesicles released by microglia and influences their action on astrocytes. Front Pharmacol 8, 910. 29321741 10.3389/fphar.2017.00910 PMC5733563

[181] Lee S , Liu W , Dickson D , Brosnan C , Berman J (1993) Cytokine production by human fetal microglia and astrocytes. Differential induction by lipopolysaccharide and IL-1 beta. J Immunol 150, 2659–2667. 8454848

[182] El-Zayat SR , Sibaii H , Mannaa FA (2019) Toll-like receptors activation, signaling, and targeting: An overview. Bull Natl Res Cent 43, 187.

[183] Long H-Z , Zhou Z-W , Cheng Y , Luo H-Y , Li F-J , Xu S-G , Gao L-C (2022) The role of microglia in Alzheimer’s disease from the perspective of immune inflammation and iron metabolism. Front Aging Neurosci 14, 888989. 35847685 10.3389/fnagi.2022.888989 PMC9284275

[184] Rodríguez-Gómez JA , Kavanagh E , Engskog-Vlachos P , Engskog MK , Herrera AJ , Espinosa-Oliva AM , Joseph B , Hajji N , Venero JL , Burguillos MA (2020) Microglia: Agents of the CNS pro-inflammatory response. Cells 9, 1717. 32709045 10.3390/cells9071717 PMC7407646

[185] Li L , Acioglu C , Heary RF , Elkabes S (2021) Role of astroglial toll-like receptors (TLRs) in central nervous system infections, injury and neurodegenerative diseases. Brain Behav Immun 91, 740–755. 33039660 10.1016/j.bbi.2020.10.007 PMC7543714

[186] Garland EF , Hartnell IJ , Boche D (2022) Microglia and astrocyte function and communication: What do we know in humans? J Leukoc Biol 16, 824888. 10.3389/fnins.2022.824888 PMC8888691 35250459

[187] Peterson PK , Hu S , Salak-Johnson J , Molitor TW , Chao CC (1997) Differential production of and migratory response to β chemokines by human microglia and astrocytes. J Infect Dis 175, 478–481. 9203678 10.1093/infdis/175.2.478

[188] Conductier G , Blondeau N , Guyon A , Nahon J-L , Rovère C (2010) The role of monocyte chemoattractant protein MCP1/CCL2 in neuroinflammatory diseases. J Neuroimmunol 224, 93–100. 20681057 10.1016/j.jneuroim.2010.05.010

[189] Andjelkovic AV , Song L , Dzenko KA , Cong H , Pachter JS (2002) Functional expression of CCR2 by human fetal astrocytes. J Neurosci Res 70, 219–231. 12271471 10.1002/jnr.10372

[190] Bettcher BM , Fitch R , Wynn MJ , Lalli MA , Elofson J , Jastrzab L , Mitic L , Miller ZA , Rabinovici GD , Miller BL (2016) MCP-1 and eotaxin-1 selectively and negatively associate with memory in MCI and Alzheimer’s disease dementia phenotypes. Alzheimers Dement (Amst) 3, 91–97. 27453930 10.1016/j.dadm.2016.05.004 PMC4941041

[191] Bettcher BM , Neuhaus J , Wynn MJ , Elahi FM , Casaletto KB , Saloner R , Fitch R , Karydas A , Kramer JH (2019) Increases in a pro-inflammatory chemokine, MCP-1, are related to decreases in memory over time. Front Aging Neurosci 11, 25. 30814948 10.3389/fnagi.2019.00025 PMC6381047

[192] Galimberti D , Fenoglio C , Lovati C , Venturelli E , Guidi I , Corrà B , Scalabrini D , Clerici F , Mariani C , Bresolin N (2006) Serum MCP-1 levels are increased in mild cognitive impairment and mild Alzheimer’s disease. Neurobiol Aging 27, 1763–1768. 16307829 10.1016/j.neurobiolaging.2005.10.007

[193] Lee W-J , Liao Y-C , Wang Y-F , Lin I , Wang S-J , Fuh J-L (2018) Plasma MCP-1 and cognitive decline in patients with Alzheimer’s disease and mild cognitive impairment: A two-year follow-up study. Sci Rep 8, 1280. 29352259 10.1038/s41598-018-19807-y PMC5775300

[194] Xia M , Qin S , Wu L , Mackay CR , Hyman BT (1998) Immunohistochemical study of the β-chemokine receptors CCR3 and CCR5 and their ligands in normal and Alzheimer’s disease brains. Am J Pathol 153, 31–37. 9665462 10.1016/s0002-9440(10)65542-3 PMC1852933

[195] Hanisch UK (2002) Microglia as a source and target of cytokines. Glia 40, 140–155. 12379902 10.1002/glia.10161

[196] Choi SS , Lee HJ , Lim I , Satoh J-i , Kim SU (2014) Human astrocytes: Secretome profiles of cytokines and chemokines. PLoS One 9, e92325. 24691121 10.1371/journal.pone.0092325 PMC3972155

[197] Bernaus A , Blanco S , Sevilla A (2020) Glia crosstalk in neuroinflammatory diseases. Front Cell Neurosci 14, 209. 32848613 10.3389/fncel.2020.00209 PMC7403442

[198] Diniz LP , Tortelli V , Matias I , Morgado J , Araujo APB , Melo HM , da Silva GSS , Alves-Leon SV , de Souza JM , Ferreira ST (2017) Astrocyte transforming growth factor beta 1 protects synapses against Aβ oligomers in Alzheimer’s disease model. J Neurosci 37, 6797–6809. 28607171 10.1523/JNEUROSCI.3351-16.2017 PMC6596548

[199] Lee Hg , Ueda M , Zhu X , Perry G , Smith MA (2006) Ectopic expression of phospho-Smad2 in Alzheimer’s disease: Uncoupling of the transforming growth factor-β pathway? J Neurosci Res 84, 1856–1861. 16998902 10.1002/jnr.21072

[200] Lian H , Litvinchuk A , Chiang AC-A , Aithmitti N , Jankowsky JL , Zheng H (2016) Astrocyte-microglia cross talk through complement activation modulates amyloid pathology in mouse models of Alzheimer’s disease. J Neurosci 36, 577–589. 26758846 10.1523/JNEUROSCI.2117-15.2016 PMC4710776

[201] Trindade P , Loiola EC , Gasparotto J , Ribeiro CT , Cardozo PL , Devalle S , Salerno JA , Ornelas IM , Ledur PF , Ribeiro FM (2020) Short and long TNF-alpha exposure recapitulates canonical astrogliosis events in human-induced pluripotent stem cells-derived astrocytes. Glia 68, 1396–1409. 32003513 10.1002/glia.23786

[202] Clarke LE , Liddelow SA , Chakraborty C , Münch AE , Heiman M , Barres BA (2018) Normal aging induces A1-like astrocyte reactivity. Proc Natl Acad Sci U S A 115, E1896–E1905. 29437957 10.1073/pnas.1800165115 PMC5828643

[203] Li K , Li J , Zheng J , Qin S (2019) Reactive astrocytes in neurodegenerative diseases. Aging Dis 10, 664. 31165009 10.14336/AD.2018.0720 PMC6538217

[204] Kirkley KS , Popichak KA , Afzali MF , Legare ME , Tjalkens RB (2017) Microglia amplify inflammatory activation of astrocytes in manganese neurotoxicity. J Neuroinflammation 14, 1–18. 28476157 10.1186/s12974-017-0871-0 PMC5418760

[205] Kraft AW , Hu X , Yoon H , Yan P , Xiao Q , Wang Y , Gil SC , Brown J , Wilhelmsson U , Restivo JL (2013) Attenuating astrocyte activation accelerates plaque pathogenesis in APP/PS1 mice. FASEB J 27, 187–198. 23038755 10.1096/fj.12-208660 PMC3528309

[206] Von Bernhardi R , Eugenín J (2004) Microglial reactivity to β-amyloid is modulated by astrocytes and proinflammatory factors. Brain Res 1025, 186–193. 15464759 10.1016/j.brainres.2004.07.084

[207] Furman JL , Sama DM , Gant JC , Beckett TL , Murphy MP , Bachstetter AD , Van Eldik LJ , Norris CM (2012) Targeting astrocytes ameliorates neurologic changes in a mouse model of Alzheimer’s disease. J Neurosci 32, 16129–16140. 23152597 10.1523/JNEUROSCI.2323-12.2012 PMC3506017

[208] Lian H , Yang L , Cole A , Sun L , Chiang AC-A , Fowler SW , Shim DJ , Rodriguez-Rivera J , Taglialatela G , Jankowsky JL (2015) NF*κ*B-activated astroglial release of complement C3 compromises neuronal morphology and function associated with Alzheimer’s disease. Neuron 85, 101–115. 25533482 10.1016/j.neuron.2014.11.018 PMC4289109

[209] Jo M , Kim J-H , Song GJ , Seo M , Hwang EM , Suk K (2017) Astrocytic orosomucoid-2 modulates microglial activation and neuroinflammation. J Neurosci 37, 2878–2894. 28193696 10.1523/JNEUROSCI.2534-16.2017 PMC6596722

[210] Tang M-Y , Gorin FA , Lein PJ (2022) Review of evidence implicating the plasminogen activator system in blood-brain barrier dysfunction associated with Alzheimer’s disease. Ageing Neurodegener Dis 2, 2. 35156107 10.20517/and.2022.05 PMC8830591

[211] Liu R-M , Van Groen T , Katre A , Cao D , Kadisha I , Ballinger C , Wang L , Carroll S , Li L (2011) Knockout of plasminogen activator inhibitor 1 gene reduces amyloid beta peptide burden in a mouse model of Alzheimer’s disease. Neurobiol Aging 32, 1079–1089. 19604604 10.1016/j.neurobiolaging.2009.06.003 PMC2888674

[212] Akhter H , Huang W-T , Van Groen T , Kuo H-C , Miyata T , Liu R-M (2018) A small molecule inhibitor of plasminogen activator inhibitor-1 reduces brain amyloid-β load and improves memory in an animal model of Alzheimer’s disease. J Alzheimers Dis 64, 447–457. 29914038 10.3233/JAD-180241

[213] Rocha SM , Cristovão AC , Campos FL , Fonseca CP , Baltazar G (2012) Astrocyte-derived GDNF is a potent inhibitor of microglial activation. Neurobiol Dis 47, 407–415. 22579772 10.1016/j.nbd.2012.04.014

[214] Lai S-W , Chen J-H , Lin H-Y , Liu Y-S , Tsai C-F , Chang P-C , Lu D-Y , Lin C (2018) Regulatory effects of neuroinflammatory responses through brain-derived neurotrophic factor signaling in microglial cells. Mol Neurobiol 55, 7487–7499. 29427085 10.1007/s12035-018-0933-z

[215] Li F , Geng X , Yun HJ , Haddad Y , Chen Y , Ding Y (2021) Neuroplastic effect of exercise through astrocytes activation and cellular crosstalk. Aging Dis 12, 1644. 34631212 10.14336/AD.2021.0325 PMC8460294

